# Supplementary material for: Hexagons all the way down: grid cells as a conformal isometric map of space
Source: PLoS Comput Biol. 2025 Feb 13;21(2):e1012804. doi: 10.1371/journal.pcbi.1012804 (PMC11841915; doi:10.1371/journal.pcbi.1012804)
Supplement: S1 Text [file pcbi.1012804.s001.pdf]

S1 Text

Loss history

In S1 Fig A, we track the conformal isometry loss (from Eq 4) over 5000 training iterations for cell counts ranging from 1 to 14. Notably, there is a stark reduction in loss, approximately ten orders of magnitude, when utilizing seven cells compared to fewer. This contrast suggests that a module of seven grid cells is a threshold for forming a conformal isometry, where fewer cells can not.

Further observations reveal that cell counts from 8 to 14 generally achieve similar losses, albeit with variations in the number of training iterations required. However, modules with 10 and 12 cells exhibit peculiar behaviours; particularly, modules with 10 cells seem unable to reach the lower loss magnitudes indicative of an accurate conformal isometry. This may hint at an inherent limitation or suboptimal configuration within the 10-cell arrangement for encoding space as a conformal isometry.

Seven colour map of hexagonal torus

S1 Fig B shows an example of the seven-colour theorem.

Toroidal topology for CI optimised models

In order to answer the question of how many cells are needed for a group of grid cells to generate a torus, we trained CI models with between one and fourteen cells. In terms of (co)homology groups computed through persistent (co)homology, a torus is characterised by having two large (persistent) one-dimensional features and one two-dimensional feature.

As one can see from S1 Fig C, which shows the sizes of these features as a function of the number of cells, the two-dimensional feature appears at three cells. At five cells there are both a two-dimensional and a one-dimensional feature, but the second two-dimensional feature is yet to appear. At six cells, there is a qualitative jump in the second one-dimensional feature, at this point the manifold has all the features necessary to characterise a torus. We therefore claim that six cells is an upper bound on the minimum amount of cells necessary for the emergence of a torus. This observation is in line with the empirical fact that one needs at least 6 PCA components to account for most of the variance in grid-cell data. A theoretical explanation for why this might happen can be found in the supplementary information of [38].

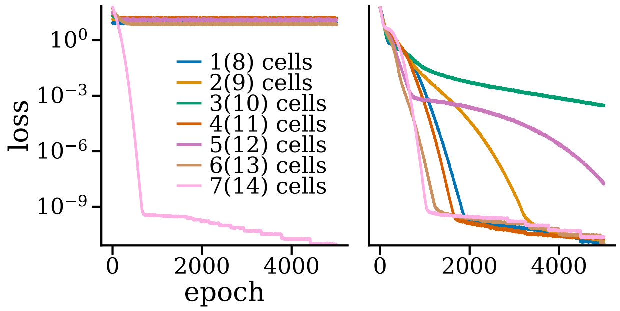

S1 Fig A. Conformal isometry loss history for 1–7 cells (left panel) and 8–14 cells (right panel). The numbers in parentheses in the legend indicate the cell count for the right panel. The y-axis range for the right panel is identical to that of the left panel.

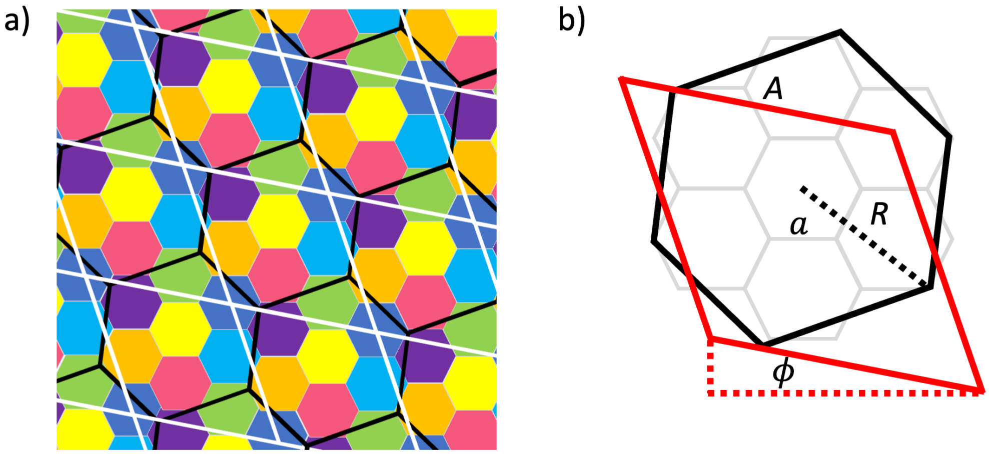

S1 Fig B. Visualisation of the 7-colour map of a flat hexagonal torus. a, b) 7 hexagons (coloured/grey), with side lengths a, optimally and recursively arranged to tile a plane, are contained in repeating, larger hexagons (black) of radius $R = \sqrt{7}a$ or rhombi (white/red) of side lengths $A = \sqrt{21}a$, tilted $\phi = \arctan\frac{\frac{\sqrt{3}}{2}a}{\frac{\sqrt{9}}{2}a} \approx 10.89$ degrees compared to the orientation of the smaller hexagons.

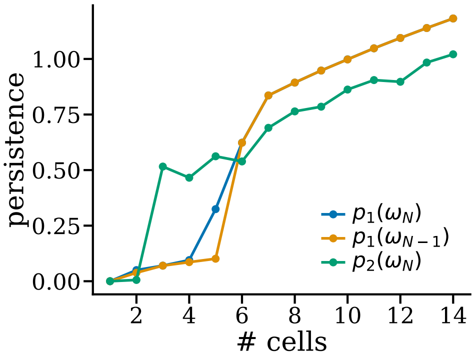

S1 Fig C. Persistence plot of the largest one dimensional feature (blue), the second largest one dimensional feature (orange) and the largest two dimensional feature (green) in a CI models with different numbers of cells.

Ripley's H sanity checks

To verify the results of the Ripley's H function presented in Fig 3i, we conduct a series of sanity checks. We start with a model of seven cells optimised for conformal isometry over 5000 iterations. From this optimised model, we infer the learned hexagonal solution and replicate it 15 times, introducing varying degrees of noise (common phase shift) to each copy.

In the left panel of S1 Fig D, we introduce normal noise with zero mean and three distinct spreads, σ, to the hexagonal solution. For a minimal spread of $\sigma = 0.01$, the Ripley's H function exhibits a highly deterministic and pronounced zig-zag pattern. This observation is facilitated by 40 permutations of noise for each spread, allowing us to include $\pm$ standard deviation error bars. The zig-zag pattern arises because, with minimal noise, so that even balls of small radius encompass most points, resulting in a higher-than-expected number of points within these balls. As the radius increases without including additional points, the function dips, reflecting a decrease in the relative expectation of points per ball. However, once the radius extends slightly beyond $r = 2/f\sqrt{21}$, two times the apothem of the hexagonal Voronoi cells (refer to 3d), it suddenly includes phases from adjacent clusters, causing a sharp uptick in the number of points within the balls.

Conversely, as the noise spread increases to $\sigma = 0.05$ and 0.15, Ripley's H function transitions to a smoother and flatter profile. This change is logical, as a larger spread of noise means that balls must be larger to encompass the more dispersed points, leading to a more gradual change between the expected and actual number of points within the balls. This smoothing effect reflects the increased randomness and dispersion of points, diluting the pronounced zig-zag pattern observed at lower noise levels.

The right panel of S1 Fig D contrasts a baseline of random uniform sampling within the unit cell (green curve) against the structured arrangement of the hexagonal solution (orange curve). Here, 15 copies of the 7-cell hexagonal solution are subjected to noise, which, unlike the left panel, is uniformly distributed across the hexagonal cell, leading to completely random centres for the copied solutions. Despite this randomness, the inherent structure of the hexagonal solution imparts a distinct spatial statistic, as evidenced by Ripley's H function. This distinction is particularly noticeable as a dip in the graph below the expected number of points for balls with a radius smaller than $r = 2/f\sqrt{21}$. Subsequently, we can see a sharp transition to a higher-than-expected number of points as the radius of the balls includes neighbouring hexagonal points. This characteristic is markedly evident in the hexagonal arrangement, where the surrounding points abruptly contribute to the count, creating a clear demarcation in the Ripley's H function. These transitions can be somewhat smoothed out for non-perfect hexagonal copies, leading to a less pronounced but still distinguishable pattern compared to a completely random phase distribution, as also observed in Fig 3i. This behaviour underscores the unique spatial signature of the hexagonal arrangement, even amidst increased randomness.

Increasing the Number of Cells with Random Phases Does Not Produce Emergent Conformal Isometry

To explore whether increasing the number of cells in a module with random, uniformly distributed phases could lead to an emergent conformal isometry (CI), we analysed modules containing various numbers of grid cells, each with randomly assigned phases. Specifically, we computed the conformal isometry score (CIS) and normalized it by the cell count (Ncells) to assess whether larger modules might inherently approximate CI.

As shown in S1 Fig E, the CIS scaled by Ncells remains stable across different module sizes, indicating that simply increasing the number of cells with random phases

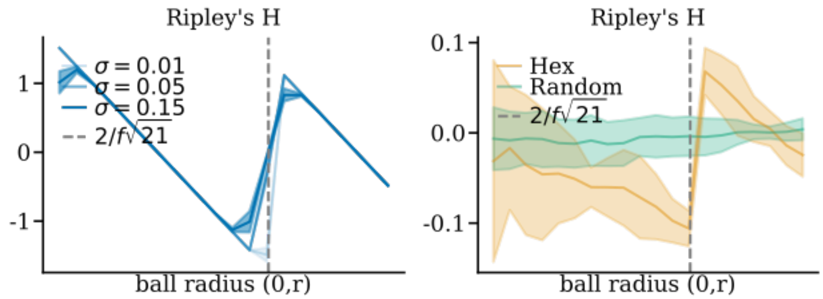

S1 Fig D. Sanity check for Ripley's H function. The left panel shows 15 copies of a module of 7 cells optimised for CI with varying degrees of common normal noise, as indicated by the legend. The dashed line at $2/f\sqrt{21}$ represents the expected distance between phases arranged as hexagons with size 1/7 of the unit cell. The right panel provides a baseline of random uniform phases, along with 15 copies of the 7-cell module optimised for CI with uniformly distributed random noise shifts.

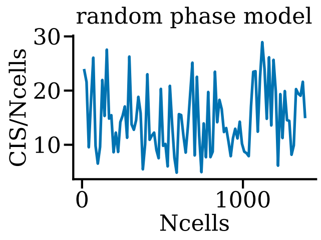

S1 Fig E. Conformal Isometry Score (CIS) Does Not Improve with More Cells in a Module with Random Phases. Plot of the conformal isometry score (CIS) normalized by the cell count (Ncells) for modules of varying sizes with random, uniform phase arrangements. Results suggest that increasing the number of cells alone does not lead to an emergent conformal isometry.

does not bring the module closer to achieving CI. This suggests that the spatial organization of phases, rather than sheer cell count, is crucial for approximating CI in grid cell networks. In other words, a specific, non-random arrangement of phases is necessary to reduce spatial distortions and approach CI properties.

These results reinforce our hypothesis that achieving CI in biological grid cells may require precise phase arrangements rather than relying on random configurations to produce emergent spatial coherence as the module size increases.

Conformal isometry in experimental grid cells

To test the conformal isometry (CI) hypothesis, we used the publicly available dataset from [38]. Fig 4 displays ratemaps from *rat_r_day1*, module 1. Similar to [53], we analyse one module from one animal, as it featured a large number of grid cells ($n = 166$ units before thresholding), long recording time in the open field compared to other animals, and a large fraction of high grid scoring units (grid score > 0.4; $n = 105$ cells after thresholding). For comparison, other modules of the same animal featured $n = 97$ and $n = 27$ high scoring cells (*rat_r_day1* modules 2 and 3, respectively). For the other comparable module (module 2), we observe minor clustering compared to noise in terms of Ripley's H, but no apparent pattern in its phase arrangement.

Ratemaps for module 1 are presented in S1 Fig Fa, alongside the corresponding grid

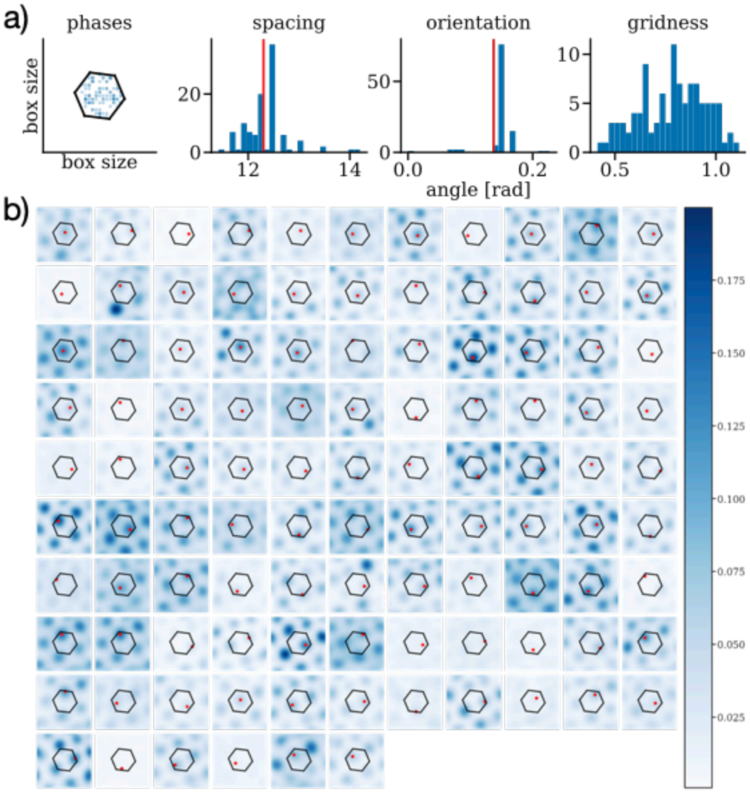

S1 Fig F. 105 Experimental Grid Cell Ratemaps from the publicly available dataset by [38]. The cells are from *rat_r_day1*, module 1. **a)** Grid statistics for the module, with the red line indicating inferred spacing and orientation used to set the unit cell parameters for the data. **b)** Ratemaps with a common colour range, indicated by the colour bar. The unit cell is superimposed, with inferred phases marked by red dots.

statistics for the module in S1 Fig Fb.

To quantify CI proximity, we computed the conformal isometry score (CIS) as defined in Eq 5. The results are shown in S1 Fig G, which also provides three baseline conditions for comparison. Statistical significance between the groups was assessed using the Mann-Whitney U test. We found significant differences between all groups ($p < 0.01$), except between the experimental data and the phase-shuffled group ($p = 0.25$, statistic $= 43.0$).

Notably, the experimental data aligns more closely with a CI than the space-shuffled baseline. However, when the ratemaps within a module were aligned to have equal and centred phases, the CIS values significantly differed from the experimental data, highlighting the importance of phase arrangements in determining CI proximity. Interestingly, when the ratemaps were randomly shifted in the x and y directions (effectively randomizing the phase arrangements) there was no significant difference between these randomized modules and the original data in terms of CI proximity. This indicates that while phase arrangement clearly influences CI proximity, the modules in these experimental data do not show a stronger CI alignment than randomly rearranging their phases.

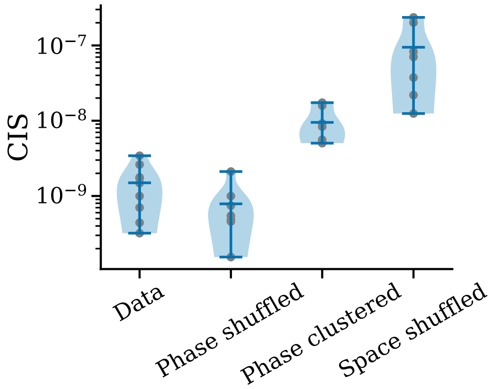

S1 Fig G. Violin Plot of Conformal Isometry Scores for Experimental and Baseline Modules for the nine modules in the publicly available dataset by [38]. The "Phase Shuffled" condition applies random shifts in the x and y directions to the ratemaps, while the "Phase Clustered" condition centres all phases. "Space Shuffled" randomly redistributes the spatial coordinates of the ratemaps.

Independence of CI solutions

Our analysis reveals that if a grid cell population $\underline{\tilde{g}}(\underline{r}) : \mathbb{R}^2 \to \mathbb{R}^N$ forms a solution to the conformal isometry loss function (Eq 4) with scale $\tilde{\sigma}$, then an extended population that includes a phase-shifted copy of this population also forms a solution, with the scale doubled to $2\tilde{\sigma}$.

To demonstrate this, consider the extended population vector defined as:

$$\underline{g}'(\underline{r}) = \begin{pmatrix} \underline{\tilde{g}}(\underline{r}) \\ \underline{\tilde{g}}(\underline{r} + \underline{\phi}) \end{pmatrix}. \tag{11}$$

The Jacobian of this extended population can be expressed as a block matrix:

$$J'(\underline{r}) = \begin{pmatrix} \tilde{J}(\underline{r}) \\ \tilde{J}(\underline{r} + \underline{\phi}) \end{pmatrix}. \tag{12}$$

This formulation allows us to derive the metric tensor of the extended population:

$$\begin{aligned} M' &= J'^T(\underline{r}) J'(\underline{r}) \\ &= \tilde{J}^T(\underline{r})\tilde{J}(\underline{r}) + \tilde{J}^T(\underline{r}+\underline{\phi})\tilde{J}(\underline{r}+\underline{\phi}) \quad \text{(independence)} \\ &= \tilde{M}(\underline{r}) + \tilde{M}(\underline{r}+\underline{\phi}) \\ &= \tilde{\sigma} I + \tilde{\sigma} I \\ &= 2\tilde{\sigma} I \end{aligned} \tag{13}$$

where we in the second step have used that a matrix $X \in \mathbb{R}^{m \times n}$ multiplied with itself $X^T X$ can be written as $(X_{1:m/2})^T X_{1:m/2} + (X_{m/2:m})^T X_{m/2:m}$.

In the fourth step, we are using that a module of grid cells that constitute a CI is independent (still a solution to CI) to a common phase shift. This can be seen by inducing a common phase shift $\underline{\phi}$ in each grid cell and rewriting it as a spatial displacement of the grid module $\underline{g}(\underline{r} + \underline{\phi})$. By virtue of CI, the metric tensor of $\underline{g}$ is constant for all $\underline{r}$, vec $\underline{r} + \underline{\phi}$. Hence, a common phase shift to a CI solution is also a CI solution. An example of this is shown computationally in Fig 3g.

Conformal scale

The conformal isometry loss is given by Eq 4. We want an extremum, which is given by $\frac{\partial \mathcal{L}}{\partial \sigma} = 0$, which yields (assuming the integrand is well behaved)

$$\sigma = \int_P \frac{G_{xx} + G_{yy}}{2|P|},$$

where $|P|$ is the volume of the region of integration, which is the parallelogram spanning the pattern unit cell. For the second derivative, we have that

$$\frac{\partial^2 \mathcal{L}}{\partial \sigma^2} = -2\frac{\partial}{\partial \sigma}\int_P G_{xx} + G_{yy} - 2\sigma d\mathbf{r} = 4|P| > 0$$

Thus the value of σ is a (local) minimum for a nonzero region of space.

Note that the parallelogram region spans all six hextants of the hexagonal unit cell of a grid pattern of radius r. However, we will rather perform integration in the unit square U. The transformation between the two is given by

$$u = \frac{1}{3r}x - \frac{y}{\sqrt{3}}$$
$$v = \frac{2}{r\sqrt{3}}y,$$

where (x, y) are parallelogram coordinates, and (u, v) square coordinates. Then, when U is the unit square,

$$|P| = \int_U |J| du dv = |J|,$$

with J being the jacobian of the transformation. For the scale σ, we have that

$$\int_P G_{ii} dx dy = \int_P \sum_{j=1}^{N} \left(\frac{\partial g_j}{\partial r_i}\right)^2 dx dy,$$

but we integrate over the full domain of each g_j, which are all of the same functional form, so every contribution is equal. Thus

$$\int_P \sum_{j=1}^{N} \left(\frac{\partial g_j}{\partial r_i}\right)^2 dx dy = N \int_P \left(\frac{\partial g_0}{\partial r_i}\right)^2 dx dy,$$

where g_0 is a grid function centred at the origin (zero phase shift). Note that this step corresponds to a coordinate shift removing the phase in each term.

Hexagonal grid Functions

A hexagonal grid function can be constructed using three plane waves, with wave vectors offset by 60 degrees. In other words, we may write a zero phase shift grid function as

$$g_0 = A \sum_{j=1}^{3} \cos(2\pi \underline{k}_j \cdot \underline{r}) + b,$$

and

$$\frac{\partial g_0}{\partial r_i} = -2\pi A \sum_j k_{ji} \sin(2\pi \underline{k}_j \cdot \underline{r}).$$

We set the wave vectors to be

$$\underline{k}_1 = (1, 0)$$
$$\underline{k}_2 = \left(\frac{1}{2}, \frac{\sqrt{3}}{2}\right)$$
$$\underline{k}_3 = \left(-\frac{1}{2}, \frac{\sqrt{3}}{2}\right),$$

so

$$\begin{aligned} G_{xx} &= A^2 \left(2\pi \sin(2\pi x) + \pi \sin\left(\pi x + \sqrt{3}\pi y\right) + \pi \sin\left(\pi x - \sqrt{3}\pi y\right)\right)^2 \\ &= 4\pi^2 A^2 \left(\sin(2\pi x) + \sin(\pi x)\cos(\pi\sqrt{3}y)\right)^2, \end{aligned}$$

where we have used the identity $\sin(u+v) + \sin(u-v) = 2\sin(u)\cos(v)$. Likewise

$$\begin{aligned} G_{yy} &= A^2 \left(\frac{2\pi\sqrt{3}}{2}\left(\sin\left(\frac{2\pi}{2}x + \frac{2\pi\sqrt{3}}{2}y\right) - \sin\left(\frac{2\pi}{2}x - \frac{2\pi\sqrt{3}}{2}y\right)\right)\right)^2 \\ &= 4\pi^2 A^2 \left(\sqrt{3}\cos(\pi x)\sin\left(\pi\sqrt{3}y\right)\right)^2, \end{aligned}$$

where we have used that $\sin(u+v) - \sin(u-v) = 2\cos(u)\sin(v)$. We thus need to integrate

$$\sigma = \frac{4\pi^2 A^2 N}{2|P|}\int_P \left(\sin(2\pi x) + \sin(\pi x)\cos(\pi\sqrt{3}y)\right)^2 + 3\cos^2(\pi x)\sin^2\left(\pi\sqrt{3}y\right) dxdy.$$

We then perform the coordinate transformation, and insert the Jacobian determinant (which cancels $|P|$), and find that the integral may be written

$$\begin{aligned} \sigma = 2\pi^2 A^2 N \int &\left(\sin\left(2\pi\left(3ru + \frac{r}{2}v\right)\right) + \sin\left(\pi\left(3ru + \frac{r}{2}v\right)\right)\cos\left(\pi\frac{3r}{2}v\right)\right)^2 \\ &+ 3\cos^2\left(\pi\left(3ru + \frac{r}{2}v\right)\right)\sin^2\left(\pi\frac{3r}{2}v\right) dudv. \end{aligned}$$

which yields

$$\sigma_{\text{hexagon}} = 3\pi^2 A^2 N$$

Square grid functions

The derivation is the same for a square grid pattern, only simpler. A square grid can be constructed using two plane waves, whose wave vectors are offset by 90 degrees. Thus

$$g_0 = A(\cos 2\pi x + \cos 2\pi y) + b,$$

and

$$\frac{\partial g_0}{\partial x} = -2\pi A \sin(2\pi x),$$

and

$$\frac{\partial g_0}{\partial y} = -2\pi A \sin(2\pi y).$$

Then

$$\sigma_s = \frac{4\pi^2 A^2 N}{2|\mathcal{U}|}\int_{\mathcal{U}} \sin^2(2\pi x) + \sin^2(2\pi y) dxdy,$$

Where $|\mathcal{U}| = 1$ is the area of the square unit cell. Then

$$\sigma_{\text{square}} = 4\pi^2 A^2 N \int_0^1 \sin^2(2\pi x) dx$$

which is just

$$\sigma_{\text{square}} = 2\pi^2 A^2 N.$$

Hexagonal vs. Square scale

Comparing hexagonal and square firing patterns, we have that

$$\frac{\sigma_{\text{hexagon}}}{\sigma_{\text{square}}} = \frac{3\pi^2 A^2 N}{2\pi^2 A^2 N} = \frac{3}{2},$$

so that for a given firing amplitude and population size, hexagonal firing patterns yield a greater conformal scale.

Proof of Injection

We consider

$$\tilde{g}_l(\underline{x}) = \frac{9}{2}\left(g_l(\underline{x}) - \frac{1}{3}\right) = \sum_{m=1}^{K} \cos\left(2\pi\, \underline{k}_m \cdot \left(\underline{x} - \underline{\phi}_l\right)\right) \quad . \tag{14}$$

similar to Eq 3 which expresses the grid field of a single grid cell characterised by the K wave vectors $\{\underline{k}_m\}$. Each grid cell l is characterised by a phase vector $\underline{\phi}_l$. In accordance with behavioural experiments, we assume a 2-dimensional world, and hence $\underline{x} \in \mathbb{R}^2$. We collate the population response of N grid cells in one N-dimensional vector $\underline{H} \in [-1,1)^N \subset \mathbb{R}^N$

$$\underline{H}(\underline{x}) = \begin{pmatrix} \tilde{g}_1(\underline{x}) \\ \tilde{g}_2(\underline{x}) \\ \vdots \\ \tilde{g}_N(\underline{x}) \end{pmatrix} \quad . \tag{15}$$

Theorem .1 (Unique Grid Cell Code)**.** *A bijective grid cell code of K wave vectors $\{k_m\}$ requires at least $N = K$ grid cells with mutually different phases $\{\underline{\phi}_l\}$.*

Proof. First, we introduce the complex formulation of the problem by using Euler's formula and taking the real part of the expression to ensure $\underline{H}$ remains real-valued

$$\begin{aligned} \tilde{g}_l(\underline{x}) &= \sum_{m=1}^{K} \mathrm{Re}\left\{\exp\left[i\,2\pi\,\underline{k}_m \cdot \left(\underline{x} - \underline{\phi}_l\right)\right]\right\} \\ &= \sum_{m=1}^{K} \mathrm{Re}\left\{\exp\left[i\,2\pi\,\underline{k}_m \cdot \underline{x}\right]\, \exp\left[-i\,2\pi\,\underline{k}_m \cdot \underline{\phi}_l)\right]\right\} \quad . \end{aligned} \tag{16}$$

Since taking the real part is a linear operation which preserves linear structures,

$$\tilde{g}_l(\underline{x}) = \mathrm{Re}\left\{\sum_{m=1}^{K} \exp\left[-i\,2\pi\,\underline{\phi}_l \cdot \underline{k}_m\right]\, \exp\left[i\,2\pi\,\underline{k}_m \cdot \underline{x}\right]\right\} \quad . \tag{17}$$

For convenience, we introduce matrix notation. Let $A \in \mathbb{C}^{N \times K}$ with $A_{l\,m} = \exp\left[-i\,2\pi\,\underline{\phi}_l \cdot \underline{k}_m\right]$ and $\underline{v} \in \mathbb{C}^K$ with $v_m(\underline{x}) = \exp\left[i\,2\pi \underline{k}_m \cdot \underline{x}\right]$. Then, we notice that Equation 17 formally expresses a matrix multiplication such that

$$\begin{aligned} \tilde{g}_l(\underline{x}) &= \mathrm{Re}\left\{\sum_{m=1}^{K} A_{l\,m}\, v_m\right\} \\ &= \mathrm{Re}\left\{(A\,\underline{v})_l\right\} \quad . \end{aligned} \tag{18}$$

Consequently, the population grid field becomes

$$\underline{H}(\underline{x}) = \mathrm{Re}\left\{A\,\underline{v}(\underline{x})\right\} \quad . \tag{19}$$

In order for $\underline{H}(\underline{x})$ to be locally bijective (one-to-one and onto), a neighbourhood $U \subset \mathbb{R}^2$ needs to exist such that for every $\underline{\tilde{H}}$ in a neighbourhood $V \subset [-1,1)^N$ exists exactly one $\underline{x} \in U$ such that

$$\underline{\tilde{H}} = \underline{H}(\underline{x}) = \mathrm{Re}\left\{A\,\underline{v}(\underline{x})\right\} \quad . \tag{20}$$

Assuming such neighbourhoods exists, Eq 14 constructs a respective $\underline{\tilde{H}}$ for each $\underline{x} \in U$. In order to show that the grid field is one-to-one, we need to provide a way of constructing a corresponding $\underline{x}$ for each $\underline{\tilde{H}}$. To do so, we need to resolve Eq 20 for $\underline{v}$. Eq 20 can be resolved iff a pseudo-inverse $A^+ \in \mathbb{C}^{K \times N}$ for A exists such that $A^+\,A = \mathbb{I}$. That is the case iff A has full rank: $\mathrm{rank}(A) = \min(K, N)$; and $\mathrm{rank}(A) = K$. Consequently, A^+ exists iff

$$\min(K, N) = \mathrm{rank}(A) = K \quad \Leftrightarrow \quad N \geq K \quad . \tag{21}$$

Consequently, the grid cell code can only be bijective if we have a response from at least K different grid cells. Because the entries in A are determined by the dot products $\underline{\phi}_l \cdot \underline{k}_m$, full rank requires at least K mutually different phase vectors $\underline{\phi}_l$. Since $\underline{\tilde{H}} = \mathrm{Re}\left\{\underline{\tilde{H}}\right\}$ and taking the real part is linear,

$$\mathrm{Re}\left\{A^+\,\underline{\tilde{H}}\right\} = \mathrm{Re}\left\{A^+\,A\,\underline{v}(\underline{x})\right\} = \mathrm{Re}\left\{\underline{v}(\underline{x})\right\} = \begin{pmatrix} \cos(2\pi\,k_1 \cdot \underline{x}) \\ \cos(2\pi\,k_2 \cdot \underline{x}) \\ \vdots \\ \cos(2\pi\,k_K \cdot \underline{x}) \end{pmatrix} \tag{22}$$

For every $Q_m = [2\pi\,k, \pi + 2\pi\,k)$ or $Q_m = [-\pi + 2\pi\,k, 2\pi\,k)$ with arbitrary $k \in \mathbb{Z}$, the cosine function is bijective on Q_m. $2\pi\,\underline{k}_m \cdot \underline{x} \in Q_m$ iff

$$k \leq \underline{k}_m \cdot \underline{x} < \frac{1}{2} + k \quad \text{or} \quad k - \frac{1}{2} \leq \underline{k}_m \cdot \underline{x} < k \quad . \tag{23}$$

For both cases exist open neighbourhoods $U_m \subset \mathbb{R}^2$ such that the respective criterion is satisfied $\forall \underline{x} \in U_m$. Consequently, the grid cell code is bijective in $\mathbb{R}^2 \supset U = \cap_{m=1}^{K} U_m$. $\square$

Calculating neural trajectory distance with the metric tensor

We can calculate local distances on the neural grid manifold model, $\underline{g}(\underline{r})$, defined by Eq 3, using the metric tensor $G(\underline{r})$. The local distance between two nearby points in this space is given by:

$$\lim_{\mathrm{d}\underline{r} \to \underline{0}} d\left(\underline{g}(\underline{r} + \mathrm{d}\underline{r}), \underline{g}(\underline{r})\right) = \sqrt{\mathrm{d}\underline{r}^\top G\left(\underline{g}(\underline{r})\right) \mathrm{d}\underline{r}}. \tag{24}$$

For an arbitrary trajectory $\underline{r}(t)$ parametrised by $t \in [0,1]$ connecting any two points $\underline{a} = \underline{r}(0)$ and $\underline{b} = \underline{r}(1)$, and a corresponding neural trajectory $\underline{g}(\underline{r}(t))$, the total distance can be computed by integrating along the trajectory:

$$|\underline{g}(\underline{r}(t))| = \int_0^1 \sqrt{\underline{r}'(t)^\top G\left(\underline{g}(\underline{r}(t))\right)\underline{r}'(t)}\,\mathrm{d}t. \tag{25}$$

From a neural encoding perspective, calculating accurate trajectory lengths from a neural trajectory would require both the neural encoding (the grid code $\underline{g}(\underline{r})$) and the metric tensor G, which describes how the grid code representation of space is distorted. This raises an intriguing question: does the brain encode not only a grid code but also a representation of the metric tensor? If so, this would imply the existence of an additional substrate responsible for encoding G, which would be a remarkable finding indeed.

Alternatively, as we have advocated in this paper, the grid code might instead act as a *conformal isometric map*. In this case, the metric tensor simplifies to a scaled identity matrix, $G = \sigma^2 I$, where $\sigma > 0$ is a constant scaling factor. The computation of neural trajectory distances then becomes significantly simpler:

$$|\underline{g}(\underline{r}(t))| = \int_0^1 \sqrt{\underline{r}'(t)^\top \sigma^2 I \underline{r}'(t)}\,\mathrm{d}t \tag{26}$$

$$= \int_0^1 \sqrt{\sigma^2 \|\underline{r}'(t)\|^2}\,\mathrm{d}t \tag{27}$$

$$= \sigma \int_0^1 \|\underline{r}'(t)\|\,\mathrm{d}t. \tag{28}$$

Which is just the (conformal) scaled distance of the physical trajectory distance. This simplification suggests that if the brain uses a conformal mapping for the grid code, distances encoded in the grid code are inherently proportional to the actual spatial distances represented by the neural trajectory. In this framework, the scaling factor σ might reflect properties such as the density of neural representation or the resolution of spatial encoding. This highlights a potential computational advantage of conformal mappings in neural systems, as the need for explicit knowledge of a metric tensor is eliminated.

Trainable Conformal Scale

To verify the scaling law in Eq 2, we trained multiple models with varying numbers of grid cells within a module to minimise the CI loss in Eq 4, treating the conformal scale σ as a trainable parameter alongside the phases. Each model was trained for 10000 training steps using gradient descent and the Adam optimiser with a batch size of 64 spatial samples drawn uniformly within the unit cell of the grid pattern. For training, we used a learning rate of 0.01 and otherwise standard optimiser parameters.

Learned scale parameters are shown in S1 Fig Ha, alongside the corresponding analytic scale. Notably, learned scale parameters match their analytic counterparts across module sizes. This suggests that the analytical conformal scale derived in Conformal scale indeed corresponds to a local minimum of the conformal isometry loss.

Note that training was more unstable when the conformal scale was left trainable. This motivated us to fix the scale parameter according to the scaling law in Eq 2 when optimising other models. However, the trainable-scale models also achieved highly uniform CI-like solutions. For reference, S1 Fig Hb shows the metric components for a trained model with 100 grid cells. The diagonal metric components are both spatially uniform (up to two decimal places) and almost equal, while off-diagonal components are near-zero everywhere.

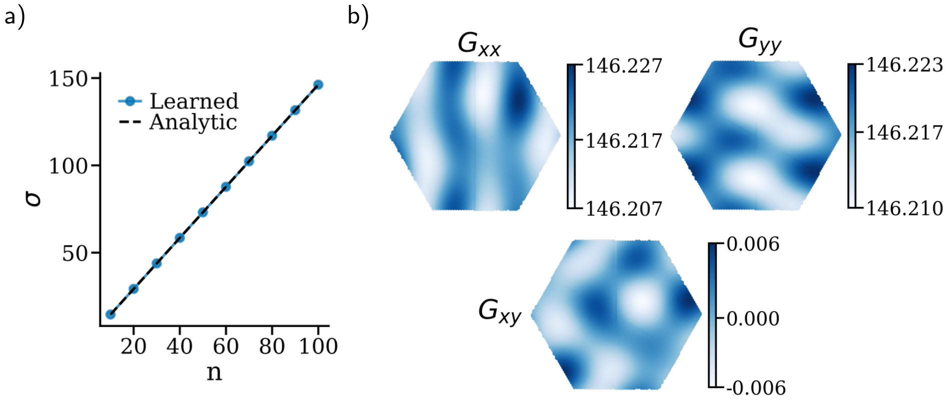

S1 Fig H. Verifying the conformal scaling law. a) Learned conformal scale (σ) as a function of the number of grid cells (n) in the module. Also indicated is the theoretically predicted scale (dashed line). b) Metric components for a module of $n = 100$ grid cells with trainable phase distribution and scale.

Grid distortions from distorted metrics

The primary focus of this work has been to study a single module of grid cells in the open field, and their properties as they relate to conformal isometry. However, in animals, grid cell firing patterns can deviate significantly from the idealised hexagonal grid for which they are known. In fact, grid patterns exhibits distortions in several scenarios, including non-symmetric geometries [40] and in response to rewards [56]. However, why such distortions arise is subject to debate [23], and is important for understanding grid cell function.

In this section, we therefore explore whether grid distortions can be understood as distortions of the metric represented by the animal. To observe more exotic grid distortions, such as those reported in [40], which extend beyond simple geometric distortions imposed by two enclosure geometries, a more expressive model is required than the static hexagonal grid pattern used throughout the rest of this work. We therefore consider a model of N cells, wherein unit responses are trainable, and formed by linear summation of radial basis functions. For a particular cell, its firing rate is then given by

$$f_i(\underline{r}) = \sum_{i}^{n_f} e^{-|\underline{r}-\underline{\mu}_i|^2}, \tag{29}$$

where $n_f = 30$ is the number of subfields allotted to each cell, $\underline{\mu}_i$ the centre location of a particular subfield, and $\sigma = 0.2$ a shared tuning width.

Using this model, we consider three distinct scenarios (see S1 Fig Ia and Fig S1 Fig Ib). The first acts as a baseline, where the set of firing functions should enforce a CI of the Euclidean metric in a square, open field arena. The second considers the same task, but learned in a trapezoidal arena, similar to [40]. The last considers the case where the learned representation is isometric to a *distorted metric*, in the same trapezoidal arena.

In this case, we consider the case were distances appear elongated along the horizontal axis, i.e.

$$G_{\text{distorted}} = \left(\frac{\alpha-\beta}{2x_0}x + \frac{\beta+\alpha}{2}\right) I, \tag{30}$$

where I is the identity, and α and β the values of the metric at $x = \pm x_0$, respectively. In our case, we take the width of the trapezoid to be $2x_0$, with $x_0 = 2$ (centred at the origin), and its height ranges from $y_0 = 2$ at the wide end, to 2α at the narrow end. We

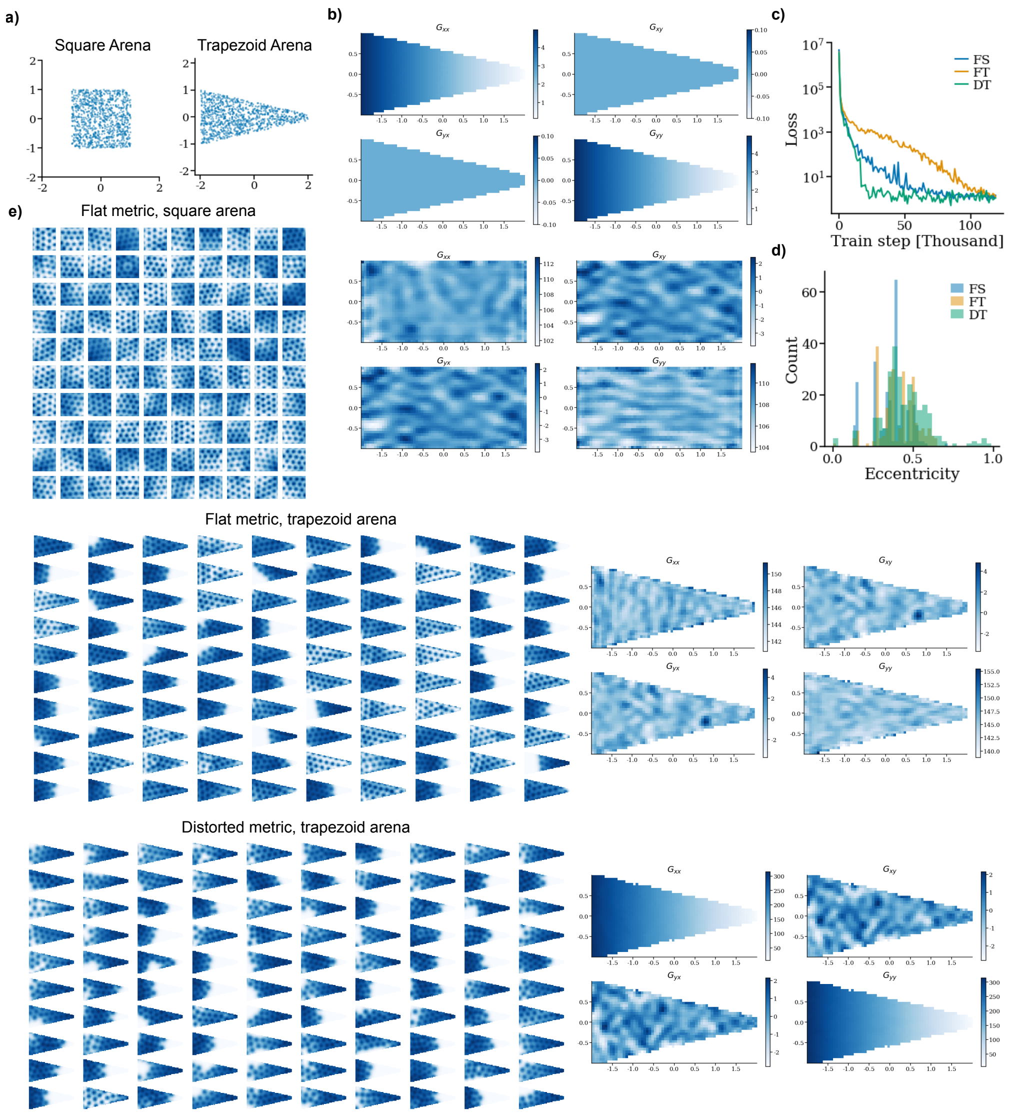

S1 Fig I. Grid distortions, geometries and non-flat metrics. a) Samples used for training models, drawn uniformly in the explored arenas **b)** The distorted metric to be learned by the model. **c)** Loss history for a baseline square environment with the flat metric (FS), a trapezoid with the flat metric (FT), and a trapezoid arena with the distorted metric in b) (DT). d) Distribution of unit pattern eccentricity, for the different models. **e)** Learned representations (left) and corresponding induced metrics (right) for each model.

set $\alpha = 0.1$, and $\beta = 5$ and the resulting trapezoidal arena and metric tensor can be seen in S1 Fig Ib.

Using the Jax and Optax python libraries [41, 42] we train models with $N = 256$ units, to minimize the squared difference between the metric induced by the representation, and a scaled target metric (given by either the flat metric, or the distorted metric), similar to Eq 4. Note that we treat the scale as a trainable parameter, as the learned representations are not guaranteed to be grid-like. We train all models using gradient descent and the Adam optimizer [64], using batches of 64 randomly and uniformly sampled points within the arena, from a training dataset of 100 000 samples. All models were trained for a total of 120 000 training steps. Uniform sampling in the trapezoidal arena was achieved by sampling uniformly from its enclosing square, and discarding points outside the trapezoid.

To evaluate the learned representations, we followed [40], and computed the eccentricity of the learned patterns. Specifically, we computed ratemaps of unit activity, and the corresponding autocorrelation matrices. To increase autocorrelogram fidelity, we evaluated ratemaps with $sigma = 0.1$. We subsequently identified the six innermost peaks of the autocorrelogram, and used direct linear least squares fitting [43, 44] of an ellipse, to determine its eccentricity.

The results are all presented in S1 Fig I. Notably, all models learn representations with low (and approximately equal) loss (S1 Fig Ic). However, the loss is far from the near-numerical-precision observed for the idealised grid patterns used elsewhere in this model, terminating at losses on the order of 10^{-1} to 1. However, the learned representations invariably qualitatively all capture the desired metric (S1 Fig Ie). In the square, flat metric case, for instance, the learned metric varies around a global scale of 107, while off-diagonal elements of the metric vary about zero (and the variance is minor, compared to the conformal scale).

Also remarkable, is the fact that the patterns learned in the flat-metric, square case, arrange in hexagonal patterns, similar to Entorhinal grid cells. Unlike the idealised setting, however, this ensemble displays multiple distinct orientations, and variations in firing rate across the environment. The flat-metric, trapezoidal case is also similar, in that most units exhibit striking, hexagonal firing patterns. However, in this case, some units display minute pattern distortions, and some also feature non-encoded regions (with no firing fields).

Distortions are even more pronounced in the distorted metric case where multiple units display irregular and distorted patterns, non-encoded regions. Furthermore, grid spacing appears to increase near the wide end of the environment, which we also observe as a slight increase in pattern eccentricity in the distorted-metric case (see S1 Fig Id).

Our findings resonate with experimental findings [40], which demonstrate that grid cell patterns become distorted in non-square arenas, and even capture their tendency to display more elliptical firing patterns. However, our work is just an initial investigation into this topic, which warrants a wider investigation. For one, we consider highly simplified trainable firing functions. Second, it is unclear whether our choice of distorted metric is an appropriate one, as multiple metrics could conceivably give rise to similar firing patterns. Third, it is unclear how a distorted metric should be interpreted: could it reflect an erroneous perception of the world by the animal? Or does it suggest that grid cells directly encode geometric information concerning the environment, by way of modelling non-flat metrics?

Regardless of interpretation, our work provides an interesting avenue for understanding grid cell pattern distortions, and in the future, one could look at ratemaps of experimental grid cells to uncover whether distortions correspond to meaningful metrics of the natural world.
