# Supplementary material for: Hexagons all the way down: grid cells as a conformal isometric map of space
Source: PLoS Comput Biol. 2025 Feb 13;21(2):e1012804. doi: 10.1371/journal.pcbi.1012804 (PMC11841915; doi:10.1371/journal.pcbi.1012804)
Supplement: Attachment — Submitted filename: 2024-Plos.pdf [file pcbi.1012804.s002.pdf]

Schøyen and colleagues investigate how the spatial phases of grid cells in the medial entorhinal cortex and other areas of the mammalian brain could be distributed to optimize for conformal isometry. The strength of the manuscript is its mathematical rigor and the arsenal of analytical tools they employ, some of them new to the field. We feel, however, that a strong weakness of the manuscript in its current form is the lack of connection with questions that are relevant from the biological perspective. In particular, at least one of the two following questions should be properly addressed by the authors. First, in what ways could conformal isometry be relevant for a module of biological grid cells, especially in the order of hundreds to thousands of grid cells presumably included in it? Second, is there any evidence from publicly available datasets that spatial phases of grid cells are optimized for conformal isometry, even if we are not yet fully convinced of its purpose? We believe that if the authors provided a convincing answer to at least one of these questions the manuscript would be suitable for publication in this journal.

Major points:

1- The relevance of conformal isometry from the perspective of neural computations in the medial entorhinal cortex is not convincing. What practical advantages would a network optimized for CI have over other phase arrangements and how can it be demonstrated? We find that the argument of energy being spent homogeneously across space is on its own rather weak and highly speculative. As far as we know, there is no evidence supporting the relevance of energy variability across space, and even if it was relevant it could be compensated by other neuronal types or different modules. Human studies reporting hexagonality in the BOLD signal (related to oxygen consumption) might be an argument against the idea of homogeneity. In any case, we believe the authors should find an argument grounded in neural computation. For example, Figure 5 shows rather disappointingly that decoding of position in CI optimized networks is similar to other networks. On top of this, most results seem to indicate that, if at all, conformal isometry is only relevant in the range of <10 cells, well below the size of grid cell modules. As a minor related point, while persistent homology is an interesting tool for analyzing population activity, the relevance of homology as an optimization criterion is also not clear from the perspective of neural computations.

2- The debate over the relevance of CI could be bypassed if the authors found that grid cells are actually CI-optimized. There are many public datasets where analyses such as the one in Figure 3i could be conducted.

3- The manuscript is written using a rather unorthodox style. An extreme example is the first phrase of section 2.3, but style should be revised throughout the manuscript.

4- Many figures lack axis labels or color codes to make them self-explanatory, and descriptions in figure captions are incomplete. An example is figure 3j but the authors should revise figures throughout the manuscript (some particular cases are indicated in minor comments, although all figures should be revised).

5- Figure 2c. Place fields are known to cluster around reward sites or borders. This phenomenon has always been interpreted as an increase in precision around interesting locations, and not as a distortion in spatial representation. Why are the authors choosing a different interpretation and how can they justify their choice?

6- Discussion. Paragraph 1. The advantage of CI-optimized networks over other arrangements in terms of spatial resolution and noise robustness is claimed here, but not clear in the results.

7- Paragraph 2. “Global distances can subsequently be computed by path-integrating …” This is presented as a consequence of CI-optimization, but results show equal performance in spatial decoding for all phase arrangements.

8- Figure 4 argues that changing the relative spatial phases of a group of grid cells can change the energy they consume to produce maps. The word ‘energy’ seems to be used in a confusing way here. If each cell uses an amount E of energy to generate a grid map, it is not clear how adjusting the relative phases might change the fact that the total energy spent is N*E.

Minor points:

- “However, their exact function remains a mistery”. This is a rather strong claim, especially taking into account the previous sentence.
- Figure 1
    - Panel d): It is not clear what colors represent on the borders of the unit cells
    - Panel e):
        - We suggest re-ordering or including titles, too many subpanels are unaccounted for. What is the color code representing in low-dimensional projections?
        - You should indicate the colors corresponding to the different homology groups. We reckon it would be better if you translated the x-axis (birth axis) to all grid cell modules.
- Figure 2:
    - We consider the analogy of the football field is not clear enough. We understand the authors use it to illustrate deviations from the identity matrix in the metric tensor in case the space is deformed from a rectangular shape. However, and since this example is really similar to the experiment presented by Krupic et al. (2015), how would the metric tensor look like, given the fact that we already know the experimental grid cell pattern obtained in such a case? Could the authors complete the analogy by making an explicit reference to grid pattern?
    - Panel (c): colormap should be indicated.
- Page 8. “Notably, the CI loss scales linearly …”. It is not clear to us what the implications are, if any.
- Figure 3:

    - Panel b): The colormap seems inadequate, tones of orange are confusing.
    - Panel c): The two orange dots for H1 are not discernable, perhaps using barcodes could help. Indicate homology groups.
    - Panel k-m): It appears to us that the difference between random and optimized networks should vanish as the number of cells increases towards biologically plausible levels. We also wonder whether in these panels white and blue cover the range from 0 to maximum or only small fluctuations. If the latter, an idea of the size of these fluctuations relative to the mean is necessary.
    - Panel l): Some solutions do not look hexagonal and it sounds to us that it is not strictly required that a sum of 7-cell solutions should be hexagonal. Could authors clarify this point and its discussion in page 9 (hexagonality is claimed to be evident)?
- The lower half of Figure 3 is not self-explanatory. Please improve the caption and the figure itself, including labels, color codes, etc. It is not clear if subpanels of l and m are matched, and if so what is the arrangement criterion.
- Figure 4 is particularly obscure and not self explanatory.
- Page 11, second paragraph. Huge refractory period and overall phrasing is a rather strange way of introducing the widely utilized approximation of neural activity by instantaneous firing rate.
- The last phrase of the same paragraph seems to trivialize what could eventually become an argument for the authors. As it is, it sounds like optimization of energy would be achieved by having no brain, and thus having some brain shows a trade-off. Perhaps the authors can show that CI-optimized networks achieve the same resolution or signal-to-noise-ratio with fewer neurons compared with random networks, but this is not clear as it is. By the way, speaking of the number of neurons is much more tangible than discussing energy, which cannot be measured, and also relates to optimizing resources.
- The paragraph about square vs hexagonal grids sounds a bit off-topic in the context of this section.
- Page 12. “Intriguingly, the linear decoding …”. It is not clear why this observation is intriguing. More generally, the only criterion that seems to be grounded on neural computations, and thus relevant, is spatial decoding.
- Page 18 (Ripley’s H function): It is not clear wether the phases φi are they selected randomly or regularly in space.
