# Supplementary material for: Hexagons all the way down: grid cells as a conformal isometric map of space
Source: PLoS Comput Biol. 2025 Feb 13;21(2):e1012804. doi: 10.1371/journal.pcbi.1012804 (PMC11841915; doi:10.1371/journal.pcbi.1012804)
Supplement: Attachment — Submitted filename: Review Hexagons all the way down.pdf [file pcbi.1012804.s003.pdf]

Reviewer #1:

The authors present an analysis of grid cells and their numbers from the perspective of injective mappings, conformal isometry, energetic considerations and attractor topology and geometry. In particular, they consider a plane-wave-based model for grid cells, and find (through computational and analytical results) the minimum number of grid cells and their particular relative phases necessary to form a conformal isometry of the traversed flat space within the neural representational space. Furthermore, they also establish that achieving such conformal isometry becomes easier with increasing number of cells in a given module.
Through their results, the authors have presented a novel approach to understanding the geometry of the neural encoding of grid cells. As such, I think their work will certainly be of strong interest to the mathematically inclined community that is interested in neural representations and grid cells. However, I have a small number of questions/comments that I feel need to be addressed before recommending their work for publication:

1) The motivation in the early introduction seems misplaced/: The authors state, "These findings indicate that grid patterns can emerge without being directly linked to path integration, raising questions to their exact function in the brain." This is not necessarily true, or at least does not follow from the findings described. Specifically (as the authors correctly note) the findings in Refs. [13-18] demonstrate path integration objectives can be achieved without the emergence of hexagonal-like grid cells. This only indicates that path integration is not directly linked to grid patterns. These results do not show grid patterns emerging without being related to path integration. Perhaps the authors intended to state that these findings indicate that path integration can be achieved without grid patterns, raising questions to the necessity and importance of the grid patterns?

Response:
We thank the reviewer for raising a valid point regarding our motivation. While questioning the role of path integration in grid cell function was part of our broader motivation, as correctly pointed out by the reviewer, this work does not provide evidence for the emergence of grid patterns independent of path integration.

On a related note, if the reviewer is interested, we explore this question in more detail in a separate study, available at https://doi.org/10.1101/2024.05.30.596577.

To more accurately reflect the scope of this article, we have revised the introduction to better align with our investigation of grid cells as a conformal isometric map of space. Rather than positioning path integration as a separate or opposing function, we now frame it as another theoretical function that grid cells may solve, potentially in conjunction with their role as a conformal isometric map.
We appreciate the reviewer's suggestion and believe this rephrasing will provide a more focused and accurate framing of our work.

2) What is being depicted in Fig. 1d? While I follow the gist of what is being demonstrated, it is unclear what is being plotted to show the population vector correlation, and how the set of ambiguous points is being determined. Some additional clarification in the caption or main text would make it easier to follow

Response:

We appreciate the reviewer's concern regarding the clarity of Fig. 1d (now Fig. 1c). To better explain the population vector correlation and the determination of ambiguous points, we have updated the figure caption as follows:

"Population vector correlation for 1, 2, and 3 cells' activity (left to right) relative to the activity at the red cross. White lines and dots highlight ambiguous points, defined as locations where the Euclidean distance between the activity vector at each location and that at the red cross is less than $\epsilon = 10^{-2}$*. For modules of 1 and 2 cells, ambiguities are present within the unit cell, while with 3 cells these ambiguities are resolved. The unit cell border colours (yellow, green, and pink) correspond to the phases from panel b) included in the population."*

Additionally, we have revised the main text to explicitly reference Fig.1c and clarify its interpretation. The updated text now reads:

"This concept is illustrated in Fig.~\ref{fig:F1-grid-cells}c, where colours represent the correlation between population activity at each spatial location and a reference point (marked by a red cross). White contour lines indicate regions where the population activity matches the reference point within a tolerance of $\|\vec{g}(\vec{r}) - \vec{g}(\vec{r}{\prime})\| < \epsilon = 10^{-2}$*. For modules with one or two grid cells, ambiguities persist within the unit cell. However, with three grid cells, these ambiguities are resolved, enabling a unique spatial representation."*

We hope these updates clarify the purpose and interpretation of Fig. 1c, addressing the reviewer's concerns. Thank you for bringing this to our attention.

3) The authors present an interesting claim relating the minimum number of cells necessary to obtain a conformal isometry, and the minimum number of colors needed for a planar graph on the torus. They relate this through the cells arranged as the vertices and center of a regular hexagon, and the corresponding regular Voronoi tessellation being 7-colorable. However, any 2d periodic lattice of activity would lead to a torus (for example, a square lattice instead of a hexagonal lattice). From the torus and it's coloring, one continues to require 7 colors, but from the perspective of achieving a CI the cells may not appear as lying on a hexagon and its center? How does one relate a general result of coloring on a torus to a specific form of achieving the torus through hexagonal patterning (rather than any other shape)? Some clarification in this regard would certainly be helpful.

Response:

We thank the reviewer for this interesting question. It is correct that the 7-color mapping theorem does not necessitate regular hexagonal tiles, only that we need at least 7 colors to prohibit similar coloring of adjacent tiles (given any tiling). As the argument goes in the text, we consider each cell as encoding a unique portion of the environment (otherwise we would have redundancy in the encoding), giving rise to a tiling of the torus. The minimal number of cells to uniquely define transitions between tiles is then 7, which we see is also the minimal number to obtain CI optimality. The relation is thus that for conformal isometry, we need to encode transitions ("delta x") in space uniquely and require 7 cells. That the tiles are hexagonally arranged is then rather that CI additionally requires encoding transitions equally, requiring regularity of the centers. Similarly this can be understood through efficient coding with each cell encodes an equal portion of the stimulus space, giving hexagonality through optimal packing.

We have now added a sentence to clarify that we're not suggesting the coloring does elicit hexagonality, only comparing the minimality of number of cells needed. It now reads:
*"*This criterion also aligns with the map-colour theorem, which specifies that at least seven regions (or "colours") are necessary to cover a torus without two adjacent regions sharing the same designation [heawood1890mapcolor]. *We note that this is similar to CI, which requires uniquely encoding displacements in space, but that the map-colour theorem does not require a hexagonal tiling, which is rather caused by CI additionally requiring* equal *encoding of displacements."*

4) A lot of the discussion in Sec. 2.5 is around the number of cells being around 6-7. However, the figure corresponding to this section (Fig. 5) is presented on a logarithmic scale and is a little hard to parse exactly what is qualitatively changing around 6-7 cells. Perhaps a zoom in around the smaller number of cells could be included as an inset of an appendix figure?

We agree with the reviewer that including a zoomed in version of the figure will help by better highlighting the behavior of these models near 6-7 cells. We have therefore added zoomed-in figures with linear scaling and have highlighted the locations where there are 6 (black) and 7 (red) cells.

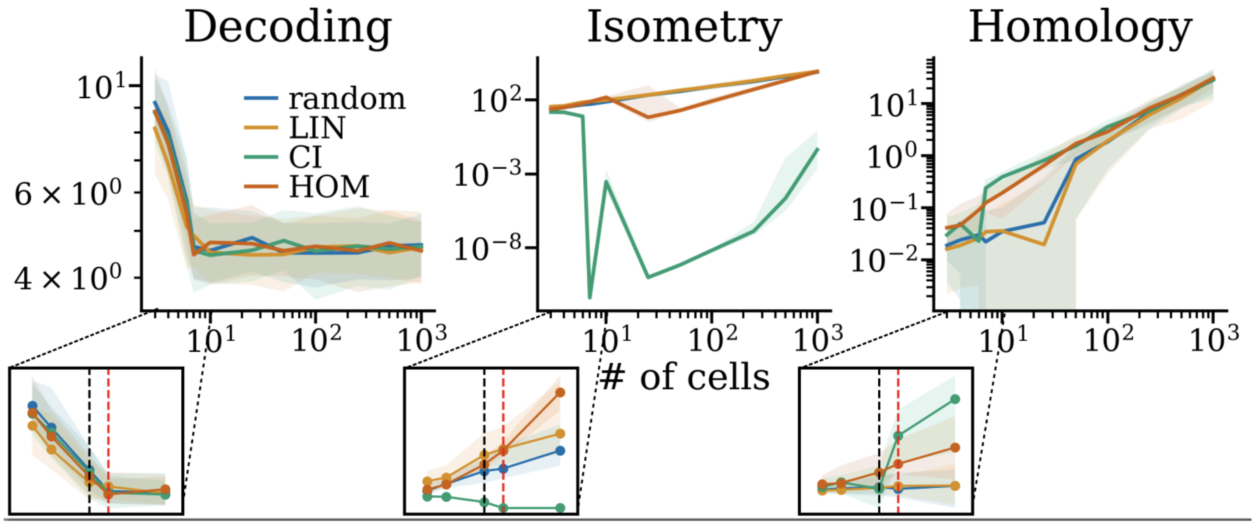

5) In Fig 5 right, why does the curve corresponding to training on the homology loss/toroidal persistence not perform the best based on the homology metric?

While we agree that this behavior of our different optimization methods is surprising, the reason for this is hard to trace as it likely relates to the complexity of the loss landscapes of these different optimization methods. Optimizing homology does lead to solutions with larger persistence, but also allows for the possibility to find other topologies (sphere with two circles attached), whereas optimizing for a flat metric seems to naturally lead to a toroidal solution with both holes being of equal size (see supplementary figure 3).

6) In the introduction, the authors state that they demonstrate that "Cartesian coordinates cannot be perfectly linearly decoded from a grid cell module". Similarly, in the discussion, they state "we found that the linear decoding of Cartesian coordinates from grid cell activity faces significant limitations". This does not seem to be stated or implied in the results section discussing linear decoding. Is the idea here that 6 grid cells are sufficient for a torus, but that linear decoding loss does not decrease until 7 grid cells and above? This seems like a weak claim, since for larger than 6 cells the decoding loss seems to be quite small across the types of models (cf. Fig. 5). The authors should clarify what is meant here.

Thank you for pointing out that this was not mentioned in the results section. We have added two sentences to the second paragraph of Section 2.6, expanding on what we mean by limitations in decoding of Cartesian coordinates from grid cell activity: “[...] Moreover, the linear decoding-optimised model generally performed poorly across other criteria, failing to outperform even the random phase baseline. This supports the notion that using a linear readout layer and decoding into Cartesian coordinates appear quite independent of the exact phase distribution. However, since both topological and geometric properties are related to the phases, more expressive non-linear decoders, like cohomological decoding \cite{desilva2011, gardner2022}, are likely to have a stronger dependence on the phases.”

7) The legend for Fig A1 seems somewhat cryptic. Do the numbers in the brackets indicate the curves in the right panel? Also, does the right panel have the same scale of the y-axis as the left panel? If so, why do some larger numbers of cells, such as the green curve (10 cells?) have such significantly higher losses?

Response:

The reviewer correctly interprets that the numbers in parentheses in the legend refer to the curves in the right panel. To clarify this, we have updated the figure caption to the following:

“Conformal isometry loss history for 1–7 cells (left panel) and 8–14 cells (right panel). The numbers in parentheses in the legend indicate the cell count for the right panel. The y-axis range for the right panel is identical to that of the left panel.”

Regarding the question of why 10 cells have a higher loss than other choices of *n* (particularly compared to smaller values like 7, 8, or 9), we currently lack a definitive answer. However, we

believe this discrepancy may be related to tiling properties and the fact that 10 is not a factor of 7, and therefore does not constitute an independent 7-cell conformal isometry (CI) solution.

The structure of the CI appears linked to the regular tiling of the unit cell. In the case of 7 cells, we observe a regular hexagonal tiling in the unit cell. However, with 10 cells, such regular tiling may not occur. To explore this hypothesis further, we include both the Voronoi decomposition and the determinant of the metric tensor for a set of 10 grid cells trained for CI with *n_train_steps*=20,000.

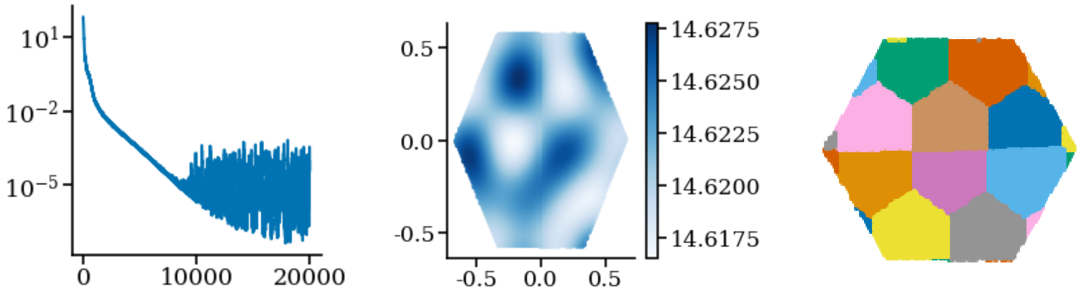

As shown, additional training helps reduce the loss, though it does not reach the same magnitude as other cell configurations (e.g., 7 cells). The determinant of the metric tensor plot reveals some spatial distortions, and the Voronoi decomposition lacks the regular tiling seen in the 7-cell hexagonal arrangement. This irregularity could account for the slower learning and the "less optimal" outcome. Specifically, it may indicate that a configuration where each cell occupies an identical partition of the unit cell does not exist for 10 cells, or if it does, it lacks the symmetry of a regular hexagon. This lack of symmetry may impact the "loss landscape," providing fewer optimal points, making random initializations farther from these optima and thus requiring longer training to converge.

Reviewer 2:

Schøyen and colleagues investigate how the spatial phases of grid cells in the medial entorhinal cortex and other areas of the mammalian brain could be distributed to optimize for conformal isometry. The strength of the manuscript is its mathematical rigor and the arsenal of analytical tools they employ, some of them new to the field. We feel, however, that a strong weakness of the manuscript in its current form is the lack of connection with questions that are relevant from the biological perspective. In particular, at least one of the two following questions should be properly addressed by the authors. First, in what ways could conformal isometry be relevant for a module of biological grid cells, especially in the order of hundreds to thousands of grid cells presumably included in it? Second, is there any evidence from publicly available datasets that spatial phases of grid cells are optimized for conformal isometry, even if we are not yet fully convinced of its purpose? We believe that if the authors provided a convincing answer to at least one of these questions the manuscript would be suitable for publication in this journal.

Response

We thank the reviewer for highlighting these points and for the opportunity to further explore the biological relevance of conformal isometry (CI) in grid cells. Below, we address both of the proposed questions, supplementing our responses with additional analyses and supporting figures.

1. **Relevance of Conformal Isometry for Large Modules of Biological Grid Cells**
   In response to the first question, we conducted a computational analysis to investigate whether modules of hundreds to thousands of grid cells with randomly arranged spatial phases could approximate CI. We extended our experiments to larger modules, considering up to 1400 grid cells with uniformly random phase distributions. For each module, we computed two metrics: (1) the mean determinant of the metric tensor normalized by the module size (ncells) to capture the extent of distortions across the module, and (2) our proposed conformal isometry score (CIS) normalized by ncells to quantify the degree to which the module approximates CI.
   Our findings, presented in the appended figure, indicate that the magnitude of distortions remains directly proportional to the module size when phases are randomly arranged. Increasing the number of cells in a module with uniformly distributed phases alone does not bring the system closer to CI, nor does it worsen CI in terms of distortions per cell. This suggests that achieving CI in a large module is not merely a consequence of increasing the cell count but may require a specific arrangement or optimization of phases. We have included a subsection “Increasing the Number of Cells with Random Phases Does Not Produce Emergent Conformal Isometry” with CIS/ncells plot in the appendix along with a similar explanation to illustrate this relationship.

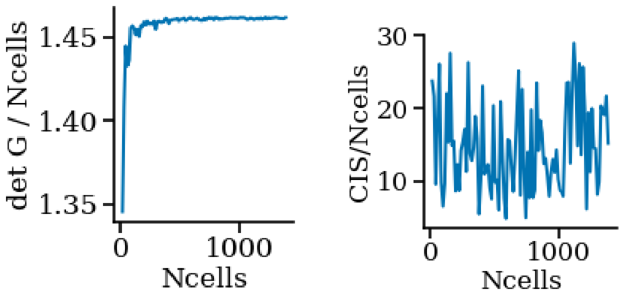

2. **Evidence of Conformal Isometry in Biological Grid Cells**

To address the second question, we analysed publicly available data from \citeauthor{gardner2022} \cite{gardner2022} and assessed whether grid cell modules exhibit properties consistent with CI. Using module 1 from \textit{rat_r_day1}, we performed a series of analyses to evaluate metric tensor components, neural-physical distance relationships, and phase distributions. These results are now included in the main text as Figure \ref{fig:F3.2-experimental-evidence}, with additional baseline comparisons and supplementary figures provided in the appendix.

Key findings include:
Metric Tensor Analysis: The experimental data demonstrates metric tensor components G_{xx}, G_{yy}, and G_{xy} that align with CI properties, where G_{xx} \approx G_{yy} and G_{xy} \approx 0 (Fig. 4 b and d)). In comparison, spatially shuffled baselines show broader, less structured distributions.
(1D) Neural-Physical Distance Relationships: Small neural distances in the experimental data correspond closely to small physical distances, as evidenced by the low variance in this regime (see low variance for small physical distance in Fig. 4 c)). This low variance in distances across space, calculated across all directions, indicates isotropic distances, a characteristic consistent with CI-like properties. In contrast, baseline comparisons with phase-clustered and spatially shuffled configurations exhibit deviations from this uniformity, highlighting their lack of CI alignment.

Cross-Module Conformal Isometry Score (CIS): We analysed the degree of conformal isometry across all nine recorded modules using the conformal isometry score (CIS). The CIS for the original data was compared to several baselines, including space-shuffled, phase-sorted, and phase-shuffled configurations. Our analysis reveals that the original data is significantly closer to a CI than the space-shuffled and phase-sorted baselines, indicating that the experimental phase arrangements are critical for supporting CI properties. However, we did not observe a significant difference between the original data and phase-shuffled configurations, as shown in the appendix. While this finding suggests that phases are important, more high-quality datasets are needed to conclusively determine whether biological grid cell modules are optimised for CI. As we have discussed in the text, some of the modules in the Gardner et al. dataset features short recording times, few high-grid-scoring units, and are recorded across multiple smaller modules with differing scales. This is also discussed in \cite{Redman_2024}. In

contrast, the module with the greatest amount of high-grid score units, and long recording time, exhibited signs of a CI, as shown in Fig. 4. We therefore argue that these analyses should be repeated with new datasets featuring larger modules and higher-resolution recordings to ascertain whether biological grid cell modules also form a CI.

Summary: While these results suggest that biological grid cells in this dataset exhibit several properties consistent with CI, they do not conclusively demonstrate optimal phase arrangements for CI. Further experimental studies with more precise data and larger-scale recordings are essential to determine the extent to which CI alignment is achieved in biological systems.

Consequences for paper: Based on the extensive additional analyses and results described above, we have revised the manuscript throughout to incorporate these findings.

Major points:

1- The relevance of conformal isometry from the perspective of neural computations in the medial entorhinal cortex is not convincing. What practical advantages would a network optimized for CI have over other phase arrangements and how can it be demonstrated? We find that the argument of energy being spent homogeneously across space is on its own rather weak and highly speculative. As far as we know, there is no evidence supporting the relevance of energy variability across space, and even if it was relevant it could bse compensated by other neuronal types or different modules. Human studies reporting hexagonality in the BOLD signal (related to oxygen consumption) might be an argument against the idea of homogeneity. In any case, we believe the authors should find an argument grounded in neural computation. For example, Figure 5 shows rather disappointingly that decoding of position in CI optimized networks is similar to other networks. On top of this, most results seem to indicate that, if at all, conformal isometry is only relevant in the range of <10 cells, well below the size of grid cell modules. As a minor related point, while persistent homology is an interesting tool for analyzing population activity, the relevance of homology as an optimization criterion is also not clear from the perspective of neural computations.

Response:
We appreciate the reviewer's concerns and the opportunity to clarify the relevance of conformal isometry (CI) in the medial entorhinal cortex. Below, we address the reviewer's concerns in detail, focusing on the computational advantages of CI, the role of geometric relationships in spatial representation, and the biological plausibility of CI in grid cells. We also reference specific revisions made to the manuscript to strengthen these points.

1. Relevance of CI in Neural Computation
A key computational advantage of CI lies in its ability to **preserve geometric relationships between locations**. CI ensures that physical distances and angles in the environment are faithfully represented in neural space, enabling accurate internal models of spatial relationships. This property is critical for spatial cognition tasks such as navigation where accuracy in the neural representation of space directly impacts performance.

In the revised manuscript, we highlight how CI supports an accurate spatial metric that could complement the role of grid cells in path integration. Specifically, CI enables a consistent representation of spatial distances, which may serve as a scaffold for integrating self-motion information with external sensory cues. This dual functionality aligns with recent normative models that optimise grid cells for both path integration and distance preservation \cite{gao2021, xu2022, xu2024, schaeffer2023}.

Revisions: To clarify this point, we have revised the abstract, introduction and discussion to emphasize the computational importance of preserving geometric relationships. We also added a subsection in the appendix that details possible impact on distance integration? "Calculating neural trajectory distance with the metric tensor"

2. Energy Homogeneity as a Secondary Benefit
While energy homogeneity across space is presented as a potential benefit of CI, we agree with the reviewer that it is not the primary justification for CI in grid cells. Instead, we argue that energy homogeneity is an emergent secondary property of a CI-optimised grid module. This property may contribute to more robust and stable neural computations, e.g. preventing localised energy spikes that could disrupt function.

To address the reviewer's concern, we have clarified that the primary relevance of CI lies in its ability to preserve geometric relationships and support an accurate spatial metric. Energy homogeneity is discussed as an auxiliary emergent advantage, rather than a central argument.
Revisions: We have revised the abstract, introduction and discussion to deemphasise energy homogeneity and focus on the computational role of CI in spatial representation.

3. Relevance of CI for Larger Modules
The reviewer notes that CI appears most relevant for modules with fewer than 10 cells, which is much smaller than typical biological modules containing hundreds to thousands of grid cells. We agree with the reviewer that a module ***can*** form a CI with as few as 7 cells. However, such a solution may be quite brittle to e.g. cell death. Moreover, we find (in Fig. 6 - previously Fig. 5) that increasing the amount of cells in a CI-module increases the conformal scale which leads to both increased spatial resolution and improvements in signal-to-noise ratio. We also highlight that random networks fail to enforce CIs, even for increasing network size, demonstrating that non-random phase arrangements are needed (see Increasing the Number of Cells with Random Phases Does Not Produce Emergent Conformal Isometry).

Revisions: We have revised Section 2.5 attempting to make these points clearer.

4. Position Decoding and Non-Linear Mechanisms
We thank the reviewer for raising this point regarding position decoding in CI-optimised networks. The observed limitations in linear decoding into Cartesian coordinates reflect the constraints of linear decoding itself rather than the relevance of CI. This supports our argument that earlier normative models, such as \cite{cueva2018}, may not fully capture grid cell

emergence due to their reliance on linear decoders. To address this, we advocate for more expressive readout mechanisms—particularly non-linear decoders, such as cohomological decoding \cite{desilva2011, gardner2022}, which may better align with the underlying structure of grid cell representations.

Revisions: We have clarified this point in the discussion, which now reads:
"We observed significant limitations in linearly decoding Cartesian coordinates from grid cell activity, suggesting that grid cells may encode space in ways incompatible with traditional linear decoding approaches. However, this does not preclude the possibility of linear decoding in alternative coordinate systems, such as polar or manifold-based representations, by downstream cell ensembles. These findings imply that relying solely on linear decoding, as in one of the earliest normative models \cite{cueva2018}, may fail to capture the true encoding mechanisms of grid cells. Instead, non-linear decoders, such as cohomological decoding \cite{desilva2011, gardner2022}, may better align with the structure of grid cell representations. These insights highlight the need for more expressive decoding methods in both biological and computational models."

5. Relevance of homology as an optimisation criterion
We thank the reviewer for their comments regarding the relevance of persistent homology as an optimisation criterion. While homology is often understood as an analytical tool, we argue that its optimisation offers valuable insights into the functional organisation of grid cell networks and the emergence of desirable properties.

Persistent homology enables us to assess the topographical organisation of population activity, identifying highly persistent structures like tori. Optimising for such structures allows us to investigate whether structural persistence serves as a sufficient condition for achieving additional functional properties, such as conformal isometry (CI), energy stability, and robust spatial decodability.

Our findings, based on cohomology optimisation, demonstrate that six cells are sufficient to encode a toroidal neural representation (see discussion in Section 4.4). This is particularly notable as it positions the minimal cell count for toroidal encoding between the numbers required to encode a unit cell (three cells) and a CI (seven cells). This result was surprising and leaves the functional significance of toroidal representations as an open question.

Revisions: This point was already addressed in the discussion, but we have revised the relevant paragraph to make it clearer. The updated text reads:

"We also investigated the feasibility of encoding spatial positions within a toroidal neural representation and linearly decoding this information from grid cell modules. While random phase arrangements typically require around 20 cells to form a torus consistently \cite{kang2021}, our findings show that only six cells are sufficient for accurate toroidal encoding. This positions the minimal set of grid cells for toroidal representation between the number required to encode a unit cell (3 cells) and the number needed for a CI (7 cells). This

raises an open question about the functional significance of toroidal representations and whether they provide computational or biological advantages in grid cell systems."

2- The debate over the relevance of CI could be bypassed if the authors found that grid cells are actually CI-optimized. There are many public datasets where analyses such as the one in Figure 3i could be conducted.
We respond to this in the first comment by the reviewer.

3- The manuscript is written using a rather unorthodox style. An extreme example is the first phrase of section 2.3, but style should be revised throughout the manuscript.
We thank the reviewer for their feedback regarding the writing style of the manuscript. In response, we have made extensive revisions throughout the manuscript to ensure the writing is more coherent and adheres to a more orthodox academic style. We believe these changes have significantly improved the readability of the manuscript.

For example, the phrasing in Section 2.3 has been revised to:
"With an understanding of the metric tensor and how phase arrangement impacts grid cell spatial representation, we explore the minimal number of grid cells required to form a conformal isometric (CI) map and the implications of increasing cell numbers within a module. [...]"
From:
"Equipped with an understanding of the metric tensor and an intuition of how phases impact grid cell representations of space, we begin our research. We explore the minimal number of grid cells required for forming a conformal iso-metric map of space and the implications of increasing the number of cells in a module.[...]"

4- Many figures lack axis labels or color codes to make them self-explanatory, and descriptions in figure captions are incomplete. An example is figure 3j but the authors should revise figures throughout the m´´

In accordance with this and other comments we have revised figure captions in an effort to make them more self-explanatory.

For example, 3j) used to be:
"Grid score analysis of learned versus random phase distributions, inferred through Gaussian kernel density estimation, plotted against varying kernel bandwidths."
Which now reads:
"*Grid score comparisons of optimised versus random phase distributions across different kernel bandwidths for Gaussian kernel density estimation (KDE). Bandwidths range up to half the unit cell radius ($r/2 = 1/3$), highlighting the hexagonality of the optimised phase arrangement."*

5- Figure 2c. Place fields are known to cluster around reward sites or borders. This phenomenon has always been interpreted as an increase in precision around interesting locations, and not as a distortion in spatial representation. Why are the authors choosing a different interpretation and how can they justify their choice?

This is a very good point, and we do agree that phase clustering near locations of interest could be a useful feature for some use-cases, such as increasing spatial resolution. However, as we describe, we hold that this could be detrimental to navigation performance. At the very least, faithful computation of traversed distances would require a representation corresponding to a metric tensor, that “undoes” the effects of distortions induced by non-CI phase arrangements (we have added a brief discussion on this, see Calculating neural trajectory distance with the metric tensor). However, that does not preclude a role of place cell clustering for other behaviorally relevant tasks (as place cells are responsive to many sources of nonspatial information), such as encoding goal locations or concepts, wherein faithful representations of distance may not be needed, or even desired. That being said, we hold that for accurate navigation, a CI solution appears both efficient and simple. However, we hope to study this subject further in the future, as this question also touches upon the related concern of distortions observed in biological grid patterns. In fact, we have added an appendix (Appendix Grid distortions from distorted metrics), wherein we *trained* a more freeform model to learn representations that are isometric to a distorted metric (and the flat metric, as a baseline). Notably, we find that metric distortions coincide with distortions of the learned grid pattern (and that a flat metric causes the network to learn hexagonal patterns), suggesting that there is a connection between isometric representation and grid cell patterns.

6- Discussion. Paragraph 1. The advantage of CI-optimized networks over other arrangements in terms of spatial resolution and noise robustness is claimed here, but not clear in the results.

We thank the reviewer for highlighting the need to better articulate the advantages of CI-optimised networks in terms of spatial resolution and noise robustness. These points were addressed in Section 2.4 (*Benefit of Many Cells*), but we recognise that they were not clearly stated. In response, we have revised Section 2.4 to explicitly outline the advantages of CI-optimised networks, including improved spatial resolution, enhanced signal-to-noise ratio, and the trade-off with energy consumption. These points are now detailed in paragraph 3 of Section 2.4, which reads:

“To quantify the change in resolution, consider a population of neurons firing with a discrete instantaneous firing rate (e.g., 0, 1, 2, ..., 200 Hz). This discretisation partitions the ambient space into distinct voxels, each representing a unique state of the population activity. As the radial size of the encoding ring grows, it intersects with an increasing number of these voxels, allowing for finer distinctions between encoded positions, as illustrated in Fig.~\ref{fig:F4-ncells}b). This increased spatial resolution enables a more precise encoding of the represented interval, thereby enhancing the system’s overall fidelity in representing spatial locations.

Second, the expanded ring also results in increased energy consumption. Here, we define "energy" as the total rate of population activity across cells, quantified by the L2 norm of the population activity. With more cells actively contributing to the encoding, the system’s overall energy expenditure rises with \(\sqrt{N/2}\) (see the larger ring radius in Fig.~\ref{fig:F4-ncells}b) as \(N \) increases). This scaling illustrates a trade-off between energy efficiency and encoding

precision, where larger cell populations achieve higher resolution at the expense of increased energy.

% signal-to-noise argument
Finally, increasing the radial size of the ring benefits the signal-to-noise ratio in the encoded variable. Assuming a fixed noise level \(\xi \) in the firing rates, the error in the encoded position is bounded by a conformal scaling factor of the error in the neural representation \cite{gao2021}. For a circle of radius \(r \), two stimuli \(s_1 \) and \(s_2 \) can be distinguished when their distance \(d(s_1, s_2) = r\theta \) exceeds \(\xi \), where \(\theta \) represents the angular separation. Thus, encoding the interval with \(N \) neurons allows for the differentiation of stimuli separated by a minimum angle of \(\xi / \sqrt{N/2} \) radians. This improvement in signal-to-noise ratio makes the encoded representation increasingly robust as the module size grows.

% SNR compared to random (asked by reviewer 2)
Importantly, CI-optimised modules achieve consistent signal-to-noise ratios across spatial locations, ensuring uniform representation of space. This contrasts with non-CI-optimised modules, where signal-to-noise ratios vary by location, leaving some regions indistinguishable while others are highly robust. Moreover, for a given spatial resolution or signal-to-noise ratio, CI-optimised networks may require fewer neurons than non-optimised or degenerate configurations, providing a more resource-efficient encoding mechanism, highlighting a potential advantage of a CI-representation."

We hope these clarifications address the reviewer's concern and clearly demonstrate the practical benefits of CI-optimised networks.

7- Paragraph 2. "Global distances can subsequently be computed by path-integrating ..." This is presented as a consequence of CI-optimization, but results show equal performance in spatial decoding for all phase arrangements.

We thank the reviewer for their fair evaluation of this sentence. Our intention was not to claim that distances can be linearly decoded. Instead, we aimed to highlight that a local preservation of distances, as provided by CI, enables the accurate representation of global distances along trajectories. This connection between local and global metrics deserved a more precise explanation and follow-up.

To address this, we have revised the discussion to include a new paragraph (paragraph 3) clarifying this point. The updated text reads:
"This local metric preservation framework extends to trajectories, ensuring that physical trajectory distances are proportional to their neural counterparts. This property implies that the grid code alone inherits all necessary metric information without requiring additional substrates to account for local distortions (i.e., a neural equivalent of the metric tensor). Consequently, neural trajectory distances computed from the grid code accurately reflect the physical distances travelled. Conversely, if distortions exist and accurate distance computations are desired, another cell ensemble encoding these physical-neural distortions—effectively acting as

a metric tensor—would need to be present. Details on trajectory distance calculations, including cases where the grid code does or does not form a CI, are discussed in \nameref{subsec:neural-trajectory-distance}."
To support this clarification, we have also included an appendix subsection titled "Calculating neural trajectory distance with the metric tensor," which provides a detailed explanation of trajectory distance calculations.

We hope these revisions address the reviewer's concern and provide a clearer explanation of the relationship between CI and distance representation.

8- Figure 4 argues that changing the relative spatial phases of a group of grid cells can change the energy they consume to produce maps. The word 'energy' seems to be used in a confusing way here. If each cell uses an amount E of energy to generate a grid map, it is not clear how adjusting the relative phases might change the fact that the total energy spent is N*E.

We appreciate the reviewer's comment and the opportunity to clarify. With our definition of energy as the L2 norm of the population activity, the energy indeed depends on the phases of the grid code. This can be easily demonstrated with two 1D sine waves: the integrated L2 norm across orientations will vary depending on their relative phases. Conversely, if energy were defined as the L1 norm, the total energy would remain invariant to phase changes, as the reviewer correctly notes.

Importantly, regardless of the norm used, the energy required at specific locations within the unit cell can vary based on the spatial phase arrangement of the grid cells. For a conformal isometry (CI), this energy is constant throughout space, allowing us to consider energy at an arbitrary point as representative of the module's energy distribution.

Figure 4d illustrates how the energy consumption at any given location in space, measured as the L2-norm of the grid population activity, scales with the module size (N) and the firing rate amplitude (A). This reflects the distribution of energy across space rather than changes in total energy.

Revisions: To clarify this, we have updated the figure caption to:
*"Population vector norm, or \textit{energy}, shown relative to the firing rate amplitude (*A*) and cell count (*N*), illustrating how energy scales with module size."*

Minor points:

- - "However, their exact function remains a mistery". This is a rather strong claim, especially taking into account the previous sentence.

We thank the reviewer for pointing out that this statement is overly strong, especially in the context of the preceding sentence. In response, we have revised the introduction to

deemphasise the contrast between grid cells' role in path integration and other potential functions. Instead, we now consider path integration as a separate and potentially joint task solved by grid cells. This revision aligns with Reviewer 1's first comment, Reviewer 2's general feedback on writing style, and this specific comment.

- - Figure 1

- - Panel d): It is not clear what colors represent on the borders of the unit cells

The lack of clarity regarding the colours on the borders of the unit cells in Panel d (now Panel c) has been addressed. The updated caption reads:

"Population vector correlation for 1, 2, and 3 cells' activity (left to right) relative to the activity at the red cross. White lines and dots highlight ambiguous points, defined as locations where the Euclidean distance between the activity vector at each location and that at the red cross is less than \epsilon = 10^{-2}*. For populations of 1 and 2 cells, ambiguities are present within the unit cell, while with 3 cells these ambiguities are resolved. The unit cell border colours (yellow, green, and pink) correspond to the phases from panel b) included in the population."*

- - Panel e):

- - We suggest re-ordering or including titles, too many subpanels are unaccounted for. What is the color code representing in low-dimensional projections?

The panels of Figure 1e have been reordered to improve clarity. Titles have been added, and the caption has been updated to explain the colours in the low-dimensional projections. The revised caption of the low-dimensional projections (f)) reads:

"Population representation of the grid cells from d). The first three plots display the population activity of the grid cells along their firing rate axes. The final plot presents the UMAP projection of the activity of 15 grid cells in three dimensions, resembling a torus. Colours represent the population vector correlation relative to the centre of the unit cell."

- - You should indicate the colors corresponding to the different homology groups. We reckon it would be better if you translated the x-axis (birth axis) to all grid cell modules.

In combination with the reviewer's comment on Figure 3c) we have changed the persistence diagrams to barcodes. We have also indicated the homology groups in the axis.

- - Figure 2:

- - We consider the analogy of the football field is not clear enough. We understand the authors use it to illustrate deviations from the identity matrix in the metric tensor in case the space is deformed from a rectangular shape. However, and since this example is really similar to the experiment presented by Krupic et al. (2015), how would the metric tensor look like, given the fact that we already know the experimental grid cell pattern obtained in such a case? Could the authors complete the analogy by making an explicit reference to grid pattern?

Thank you for raising this issue. While we initially wanted to consider only the flat-metric, open-field case due to its simplicity, we agree that this point is sufficiently important to warrant

further investigation. We therefore added a Section to the appendix (see Appendix: Grid distortions from distorted metrics) wherein we train a more freeform model, to learn firing patterns that are isometric to a distorted metric in a trapezoidal arena (and a CI of flat space, as a baseline). Notably, the distorted metric induces distortions in the grid patterns, thus (hopefully) addressing this concern, and providing an interesting interpretation of these distortions in biological cells.

- - Panel (c): colormap should be indicated.
We have now added a colorbar.

- - Page 8. “Notably, the CI loss scales linearly ...”. It is not clear to us what the implications are, if any.
The implications of this statement may not have been sufficiently clear in the original text. The linear scaling of the CI loss demonstrates that CI phase solutions are independent. This means that combining multiple CI modules does not result in a disproportionate loss increase; instead, the losses simply add together. This property highlights the modularity of CI solutions, allowing combinations of smaller modules to form valid configurations.

To clarify this, we have revised the paragraph in Section 2.3 to:
“Further, we explore phase interdependencies by moving a single phase while holding others fixed, which shows the collective role of phases in achieving CI (Fig.\ref{fig:F3-phase-distribution}h). Analytically, we find that CI solutions are independent, allowing the combination of multiple solutions to form new, valid configurations (see \nameref{subsec:CI-independence}). Computationally, this is verified by generating a module of 15 randomly phase-shifted copies of the hexagonal solution (Fig.\ref{fig:F3-phase-distribution}a), with the resulting phases and their kernel density estimates (KDEs) depicted in Fig.~\ref{fig:F3-phase-distribution}k). The empirical loss scales linearly with the number of copies, further supporting solution independence. Together, these analyses affirm that the seven-cell hexagonal phase arrangement is not simply a product of chance but rather represents an optimal solution. Although the specific arrangement of phases is crucial, the overall positioning of the hexagonal solution is subject to arbitrary phase shifts, suggesting a form of positional invariance. This independence implies a broad landscape of potential CI solutions, potentially allowing combinations of “prime” solutions, like factors of seven cells.”

- - Figure 3:
- - Panel b): The colormap seems inadequate, tones of orange are confusing.
We agree that our choice of color palette renders the colors of the toroidal projection a bit difficult to discern. However, we hope the reviewer will appreciate that we chose seaborns color palette “colorblind” to be as inclusive as possible to a wide audience.

- - Panel c): The two orange dots for H1 are not discernable, perhaps using barcodes could help. Indicate homology groups.

The two orange dots for H_1 were indeed difficult to discern. Following the reviewer's suggestion, we have updated the manuscript to use barcodes for better visualisation, with homology groups explicitly indicated in the figure.

- - Panel k-m): It appears to us that the difference between random and optimized networks should vanish as the number of cells increases towards biologically plausible levels. We also wonder whether in these panels white and blue cover the range from 0 to maximum or only small fluctuations. If the latter, an idea of the size of these fluctuations relative to the mean is necessary.

The first question regarding whether the difference between random and optimised networks vanishes with large N has been addressed in detail in our response to the first comment by the reviewer.

Panel k:
We have updated the figure to include a colorbar for clarity. Additionally, the bandwidth of the kernel density estimate (KDE) has been adjusted from 0.2 to 0.1, and the integral over the unit cell is normalised to 1, yielding a probability distribution. The figure caption has been updated to reflect these changes:

"Kernel density estimate (KDE) of the 15 replications of the phase solution from panel a), each with an additional random phase shift. Bandwidth for KDE is set at 0.1, with the resulting grid score shown in the title. The KDE is normalized within the unit cell to integrate to unity."

Panels l and m:
For the KDEs in panels l and m, we provide the standard deviations over the unit cell in the figure caption to indicate the scale of fluctuations. Adding individual colorbars to each plot would require significant figure space, which we believe would detract from visual clarity. The updated captions are:

"\textbf{l)} (top) KDE visualisation of optimised phase distributions overlaid with phase locations, with titles indicating grid scores. Four bandwidths were selected: r/20, 5r/20, 10r/20, *and* 15r/20. *Standard deviations for the KDEs are* (10^{-1} \cdot 3.17, 10^{-2} \cdot 1.95, 10^{-3} \cdot 2.77, 10^{-6} \cdot 5.93)*, reflecting the KDE colour variability across bandwidths. All KDEs are normalized to have a unit integral. (bottom) Autocorrelograms of the KDEs confirm hexagonal spatial periodicity."*

"\textbf{m)} KDEs for random phase distributions at the same bandwidths as panel l), providing a baseline comparison. KDE standard deviations are (10^{-1} \cdot 3.81, 10^{-1} \cdot 1.25, 10^{-2} \cdot 4.51, 10^{-3} \cdot 6.50)*."*

- - Panel l): Some solutions do not look hexagonal and it sounds to us that it is not strictly required that a sum of 7-cell solutions should be hexagonal. Could authors clarify this point and its discussion in page 9 (hexagonality is claimed to be evident)?

We acknowledge that not all KDEs in panel l appear hexagonal, and we agree that this is not strictly required. Instead, our point is that for a specific range of KDE bandwidths, hexagonality becomes evident. This is supported by the grid score statistics shown in panel j, which indicate a high grid score for the same module of 100 grid cells optimised for CI over a range of KDE bandwidths.

In panel l, we select four KDE bandwidths to provide a visual representation of the statistics summarised in panels i and j. To clarify this, we have updated the figure caption to be more specific about the KDE bandwidths used in panels i–m, ensuring the connection between visualisations and statistical results is explicit.

- - The lower half of Figure 3 is not self-explanatory. Please improve the caption and the figure itself, including labels, color codes, etc. It is not clear if subpanels of l and m are matched, and if so what is the arrangement criterion.

We have revised the caption for the lower half of Figure 3 to provide clearer explanations of the subpanels, including labels, colour codes, and their arrangement criteria. Additionally, we ensured that the relationship between subpanels l and m is explicitly stated, clarifying the matching and arrangement criteria.

- - Figure 4 is particularly obscure and not self explanatory.

We acknowledge that Figure 4 (now Figure 5) was not sufficiently self-explanatory. In response, we have extensively revised the figure caption to improve clarity and ensure it is self-contained. Additionally, we have made minor additions to the corresponding paragraphs in the text to better contextualise the figure.

- - Page 11, second paragraph. Huge refractory period and overall phrasing is a rather strange way of introducing the widely utilized approximation of neural activity by instantaneous firing rate.

We appreciate the reviewer’s feedback and agree that the reference to the refractory period was unnecessarily confusing. To clarify our main point—that ambient space can be partitioned into voxels when approximating firing rates by discrete states—we have revised the text. The updated paragraph now reads:

“To quantify the change in resolution, consider a population of neurons firing with a discrete instantaneous firing rate (e.g., 0, 1, 2, ..., 200 Hz). This discretisation partitions the ambient space into distinct voxels, each representing a unique state of the population activity. As the radial size of the encoding ring grows, it intersects with an increasing number of these voxels, allowing for finer distinctions between encoded positions, as illustrated in Fig.~\ref{fig:F4-ncells}b). This increased spatial resolution enables a more precise encoding of

the represented interval, thereby enhancing the system's overall fidelity in representing spatial locations."

- - The last phrase of the same paragraph seems to trivialize what could eventually become an argument for the authors. As it is, it sounds like optimization of energy would be achieved by having no brain, and thus having some brain shows a trade-off. Perhaps the authors can show that CI-optimized networks achieve the same resolution or signal-to-noise-ratio with fewer neurons compared with random networks, but this is not clear as it is. By the way, speaking of the number of neurons is much more tangible than discussing energy, which cannot be measured, and also relates to optimizing resources.

We appreciate the reviewer's comment regarding the last phrase of the paragraph. To address this, we have revised the text to emphasise how CI-optimised networks achieve consistent signal-to-noise ratios across spatial locations. This uniformity ensures that CI-optimised networks avoid regions with low distinguishability, unlike non-optimised modules where signal-to-noise ratios vary spatially. Additionally, we now highlight that CI-optimised modules can achieve the same spatial resolution or signal-to-noise ratio with fewer neurons compared to non-optimised networks, providing a more tangible and resource-based argument for the benefits of CI.

The revised paragraph reads:

"Finally, increasing the radial size of the ring benefits the signal-to-noise ratio in the encoded variable. Assuming a fixed noise level \xi in the firing rates, the error in the encoded position is bounded by a conformal scaling factor of the error in the neural representation \cite{gao2021}. For a circle of radius r , two stimuli s_1 and s_2 can be distinguished when their distance d(s_1, s_2) = r\theta exceeds \xi , where \theta represents the angular separation. Thus, encoding the interval with N neurons allows for the differentiation of stimuli separated by a minimum angle of \xi / \sqrt{N/2} radians. This improvement in signal-to-noise ratio makes the encoded representation increasingly robust as the module size grows.

Importantly, CI-optimised modules achieve consistent signal-to-noise ratios across spatial locations, ensuring uniform representation of space. This contrasts with non-CI-optimised modules, where signal-to-noise ratios vary by location, leaving some regions indistinguishable while others are highly robust. Moreover, for a given spatial resolution or signal-to-noise ratio, CI-optimised networks may require fewer neurons than non-optimised or degenerate configurations, providing a more resource-efficient encoding mechanism. This highlights the potential advantage of CI as a framework for neural representations, balancing precision and efficiency."

We hope this revision addresses the reviewer's concern and provides a clearer and more robust justification for the benefits of CI-optimised modules.

- - The paragraph about square vs hexagonal grids sounds a bit off-topic in the context of this section.

We understand the reviewer's concern regarding the inclusion of the square versus hexagonal grid comparison in this section. However, we included this analysis both as a baseline and to highlight a key advantage of hexagonal solutions. Previous work, such as Mathis et al. (2015), has also compared these patterns, and we now explicitly cite this reference in the revised text to contextualise our analysis.

The updated text reads:

"For comparison, and in line with the analysis in \cite{mathis2015}, we also derive the conformal scale for a square grid pattern, σ_{square} *, and find that the ratio* $\sigma_{\text{hexagon}} / \sigma_{\text{square}} = 3/2$ *. This indicates that the hexagonal pattern's scale—and therefore the surface area of the encoding manifold—is 50% larger than that of a module of cells with a square pattern at the same firing rate amplitude,* A *, and cell count,* N *. Consequently, a module with a hexagonal grid pattern exhibits enhanced spatial resolution and improved noise robustness compared to a similar module with a square pattern. Fig.~\ref{fig:F4-ncells}c) visualises this relationship by plotting the conformal scale for the hexagonal pattern,* σ_{hexagon} *, relative to cell count and firing rate, with light blue contour lines providing a reference for the square grid's scale,* σ_{square} *. This comparison highlights the efficiency of the hexagonal firing pattern in grid cells, supporting its role as an optimal configuration for spatial encoding."*

- - Page 12. "Intriguingly, the linear decoding ...". It is not clear why this observation is intriguing. More generally, the only criterion that seems to be grounded on neural computations, and thus relevant, is spatial decoding.

While linear decoding to Cartesian coordinates may not be directly behaviourally relevant, it is a useful criterion for evaluating models of grid cells and their decoding mechanisms. For instance, normative models may assume or implement linear decoding, as in \cite{cueva2018}, to assess how well grid cells represent spatial information. Additionally, decoding from experimental grid cells typically requires explicit assumptions about the readout mechanism, and linear decoding can serve as a baseline for comparison.

As previously discussed (see our response to Reviewer #3, Point 4), our findings suggest significant limitations in linear decoding, particularly into Cartesian coordinates. These limitations highlight that grid cells may encode spatial information in ways incompatible with traditional linear readout methods. Consequently, our results advocate for more expressive decoders, such as non-linear or cohomological decoding \cite{desilva2011, gardner2022}, both in experimental analyses and in computational models. This insight not only informs the development of more accurate decoding strategies but also underscores the need to consider alternative coordinate systems (e.g., polar or manifold-based representations) when interpreting grid cell activity. We have appended the following text to outline this more clearly:

"This supports the notion that using a linear readout layer and decoding into Cartesian coordinates is quite independent of the exact phase distribution. However, since both

topological and geometric properties are related to the phases, more expressive non-linear decoders, like cohomological decoding \cite{desilva2011, gardner2022}, are likely to have a stronger dependence on the phases."

- - Page 18 (Ripley's H function): It is not clear wether the phases φi are they selected randomly or regularly in space.

In the methods section, we provide a general description of the Ripley's H function. For the analysis presented in Fig.~\ref{fig:F3-phase-distribution}i), we use 100 randomly and uniformly sampled phases within the unit cell as a baseline and compare these to 100 phases optimised for a conformal isometry (CI).

To clarify this in the manuscript, we have added the following sentence to the description of the Ripley's H function:

"For the analysis in Fig.~\ref{fig:F3-phase-distribution}i), we use 100 randomly and uniformly sampled phases within the unit cell as a baseline, and compare them to 100 phases optimised for a conformal isometry."

Reviewer #3:

The manuscript by Schoyen and colleagues explores the hypothesis that grid cells in the brain form a conformal isometric map, i.e., they preserve angles and distances while mapping the external world. Using a relatively simple framework and model, they demonstrate that at least seven grid cells with phases arranged in a hexagonal pattern are necessary to achieve a conformal isometry. Increasing the number of grid cells improves spatial resolution but also increases energy consumption. They also comment on the feasibility of 2D decoding of spatial position from grid cells.

The manuscript is overall interesting and well-written. The concepts and intuition have been well introduced. Overall, I have some major conceptual questions and suggestions for the authors.

1. While the authors have commented and referenced the effect of environmental boundaries in distorting the grid (page 15), this experimental result has not been sufficiently discussed and modeled in the light of their results.

Several studies showing grid cells in trapezoids (Krupic et al, 2014), 3D space (Ginosaur et al, 2014), 1D space (Domnisoru et al, 2013) do not have regular hexagonal geometry, but are rather distorted. Moreover, grid cells respond in various ways when animals are performing task or when features of the environment are modified (Peng et al, 2023, biorxiv, Diehl et al, 2016, Boccara et al, 2019 and many more), modulating their rates as well as translocating towards goals.

The vast majority of experimental studies demonstrate that under regular conditions of navigation, grid cells do not form a conformal isometric map. The authors must address this discrepancy between their predictions and observed experimental results.

We appreciate the reviewer's insightful comments regarding the discrepancies between our predictions and experimental observations of grid cell activity in more complex or constrained environments. We acknowledge that our current model is highly simplified, focusing on a single module of grid cells operating in an open field with regular and symmetric firing fields. This design allows us to clearly demonstrate how grid cells, in their most basic configuration, are naturally inclined to form a conformal isometric (CI) map.

However, as the reviewer notes, grid cells exhibit distortions under various experimental conditions, including in non-Euclidean spaces (e.g., trapezoids \cite{krupic2014} and 3D spaces \cite{ginosar2023}) and during specific tasks \cite{peng2023, diehl2016, boccara2019}. These distortions likely arise from additional degrees of freedom not considered in our main model. However, as this was noted by another reviewer, and we agree that this is a sufficiently important concern, that we performed a supplementary analysis, wherein we train a more freeform model to learn distorted metrics (Appendix "Grid distortions from distorted metrics"). Notably, training this model to learn a CI yielded hexagonal grid patterns, whereas representing a distorted metric, induced distortions in the learned patterns.

We believe that CI-based models could serve as a framework for understanding both symmetric and non-symmetric grid representations. It is plausible that some grid cell modules preserve the geometric properties of physical space (consistent with CI), while others distort this representation based on the animal's internal state or goals. Thus, our main model may accurately describe the subset of symmetric grid modules, while other mechanisms may be responsible for non-symmetric distortions, such as distortions of the represented metric. However, more research into this question is warranted, but our preliminary findings point to a connection between conformal isometries and grid patterns, while pattern distortions can arise from grid cells being isometric to distorted metrics.

To further address these points, we have also expanded our discussion.

"While our model offers valuable theoretical insights, it remains simplified relative to the biological grid cell system and does not capture all experimentally observed phenomena, such as multimodularity \cite{stensola2012}, pattern distortions \cite{krupic2015} and goal-directedness \cite{boccara2019}. We have provided preliminary computational evidence that grid distortions can be understood as distortions of the represented metric (see Appendix \ref{subsec:geometric_distortions}). However, the factors that determine how the represented metric distorts remain unknown.

Furthermore, we have only explored one geometry, so future works could extend this to include environments with other geometries or incorporate salient landmarks, potentially explaining a broader class of observed grid distortions \cite{derdikman2011, ginosar2023, wen2023, boccara2019, krupic2015}. Future work could also involve distinct grid cell modules, some preserving the geometric properties of physical space, while others distort space based on the animal's internal state or behavioural goals.

How representing a distorted metric impacts navigation performance, is also an unanswered question: In the case of grid cells representing a distorted metric as discussed in \nameref{subsec:neural-trajectory-distance}, accurate computation of traversed distances could require translating between the distorted representation and undistorted representation, possibly necessitating an additional neural ensemble to do so. Additionally, exploring how CI principles apply to multiple interacting modules, path integration, or mapping more complex spaces \cite{killian2012map, aronov2017} could deepen our understanding of how grid cells flexibly and efficiently encode space."

2. Similarly, the authors should comment on how this framework applies to conjunctive grid cells in the entorhinal cortex. Do they propose that only the select, non-conjunctive grid cells (those forming the toroid as reported in Gardner et al, 2022) are the ones that form the CI? More broadly, how do they envision this network integrating within the circuits in the entorhinal cortex?

To address how this framework integrates within entorhinal cortex circuits, we envision a modular organisation, where non-conjunctive grid cells form the backbone of a spatial metric

(CI), and conjunctive grid cells provide adaptive, task-specific enhancements. This modular perspective aligns with findings from Gardner et al. (2022), which suggest that distinct grid cell modules may serve specialised functions. However, further research is needed to explore how these modules interact and whether conjunctive grid cells can also approximate CI properties under specific conditions.

To address this, we have added the following paragraph to the discussion:
“Our framework primarily focuses on symmetric, non-conjunctive grid cells as the substrate for a conformal isometric (CI) map. Conjunctive grid cells, such as those encoding combinations of spatial and head direction information \cite{sargolini2006}, may also contribute to spatial encoding, but likely through more complex interactions. It is plausible that non-conjunctive grid cells provide the foundational distance-preserving CI representation, while conjunctive grid cells integrate task-relevant variables, such as orientation or behavioural goals. This division of labour suggests a modular organisation within the entorhinal cortex, where distinct grid cell modules specialise in different aspects of spatial cognition. Future research could explore whether conjunctive grid cells are capable of approximating CI properties independently, or whether they rely on non-conjunctive modules to preserve spatial consistency.”

3. The 2D decoding results are interesting, but somewhat underexplored. “Furthermore, we found that the linear decoding of Cartesian coordinates from grid cell activity faces significant limitations, suggesting a potential mismatch between traditional linear decoding models and the actual grid cell encoding mechanisms.” Can the authors expand on this? Perhaps I misunderstood, but if the grid cell network preserves distances, wouldn’t one expect that linear decoding would be effective for estimating Cartesian coordinates? Are there alternative decoding approaches that the authors can recommend?
We appreciate the reviewer’s comment and the opportunity to clarify the limitations of linear decoding in the context of grid cell activity. While a conformal isometry (CI) preserves local distances and angles, this does not necessarily imply that Cartesian coordinates can be effectively decoded through a linear readout. The mismatch arises because grid cell activity encodes space (Cartesian coordinates) through a nonlinear (sum of plane waves) manner which is non-linearly invertible.

To address this, we have expanded the discussion to highlight the need for more expressive decoding approaches. Specifically, we note that linear decoding may still work in alternative coordinate systems, such as polar or manifold-based representations, and that non-linear decoders, such as cohomological decoding \cite{desilva2011, gardner2022}, could better leverage the topological and periodic structure of grid cell representations.

The revised text in the discussion reads:
“We observed significant limitations in linearly decoding Cartesian coordinates from grid cell activity, suggesting that grid cells may encode space in ways incompatible with traditional linear decoding approaches. However, this does not preclude the possibility of linear decoding in alternative coordinate systems, such as polar or manifold-based representations, by downstream cell ensembles. These findings imply that relying solely on linear decoding, as in

one of the earliest normative models \cite{cueva2018}, may fail to capture the true encoding mechanisms of grid cells. Instead, non-linear decoders, such as cohomological decoding \cite{desilva2011, gardner2022}, may better align with the structure of grid cell representations. These insights highlight the need for more expressive decoding methods in both biological and computational models."

We hope this explanation clarifies our findings and the rationale for exploring alternative decoding strategies.

4. I have some objection to their distinction between grid cells performing path integration and grid cells as a metric for space. The entire manuscript is written as if these are contrasting viewpoints, while these are quite complementary. Perhaps the authors can clarify the major differences in their view between these concepts?

We appreciate the reviewer's comment and agree that path integration (PI) and conformal isometry (CI) are complementary rather than opposing functions. Grid cells may use PI to update position based on self-motion while simultaneously encoding a spatial metric via CI. By preserving local distances and angles, CI could enhance the accuracy of PI, ensuring that updates are geometrically consistent with the environment.

As noted in our response to Reviewer #1, we have revised the introduction to better align with our investigation of grid cells as a conformal isometric map of space rather than positioning path integration as a separate or opposing function.
