# Supplementary material for: Hexagons all the way down: grid cells as a conformal isometric map of space
Source: PLoS Comput Biol. 2025 Feb 13;21(2):e1012804. doi: 10.1371/journal.pcbi.1012804 (PMC11841915; doi:10.1371/journal.pcbi.1012804)
Supplement: Attachment — Submitted filename: Hexagons all the way down PLOS review response v2.pdf [file pcbi.1012804.s004.pdf]

We sincerely thank the reviewers for their thoughtful feedback on our manuscript. Below are our detailed responses to Reviewers comments:

Reviewer #1 comment:
I am still unclear on the claim being made in relation to linear decoding of Cartesian coordinates from grid cell activity. Since the linear decoding loss is quite small for anything more than 6–7 cells, I do not know if it is fair to state "We observed significant limitations in linearly decoding Cartesian coordinates from grid cell activity, suggesting that grid cells may encode space in ways incompatible with traditional linear decoding approaches". I do not follow why the authors believe that there is some fundamental incompatibility --- perhaps some additional clarification could help.

Response:
We wish to thank the reviewer for giving us the opportunity to clarify our statement regarding linear decoding. We have now added a simple argmax decoder, described in section 2.6 that outperforms linear decoding and improves with an increasing number of cells on populations with a random phase distribution; this can be seen in the now updated figure 6. In addition, we have modified the sentence in question to make it clear that linear decoding is limited relative to the simple non-linear decoder in question.

Reviewer #2, Comment 1:
We suggest that the authors revise the color coding for the tensor metric plots to ensure that the colors used for these measurements do not overlap with the colors used for different types of optimizations. Several axis labels lack units (for example, 'physical distance' in Fig. 4d), and some axes lack labels.
Response:

- Thank you for this observation. Unfortunately, we are not entirely certain which plots are referred to as "tensor metric plots" or how the colors overlap with other plots. If the reviewer can provide further clarification, we would be happy to revise this accordingly.
- We appreciate the note on missing axis labels. We have added units to the relevant axes in Figure 4. For the other figures, the values are dimensionless, which is why units are omitted.

Reviewer #2, Comment 2:
Figure 1: Including a color bar that indicates the minimum and maximum correlation values directly in the figure, instead of describing the color scheme in the caption, would make the figure more self-explanatory and easier to interpret.
Response:
We thank the reviewer for this suggestion. While we agree that including a color bar can often enhance clarity, in this case, the population vector correlations are dimensionless, with the intent being to highlight (dis)similarity between spatial locations for 1, 2, and 3 cells. Since the color bar range would provide little additional insight, we have chosen to retain the current design and description in the caption.

Reviewer #2, Comment 3:

Figure 4. The new figure adds depth to the article, but the plots could be clearer. In panel d, we suggest finding a way to avoid the overlap between histograms, as some of them are hidden in the background. In panel c, we suggest focusing on the linear part supporting the claims. We also suggest clarifying what is meant by “spatially shuffled” in the figure caption.
Response:

- Thank you for these suggestions. Regarding the overlap in histograms in panel d, we note that this overlap reflects the underlying data distribution. Any attempt to separate the histograms visually (e.g., by offsetting them) might compromise the interpretability of the actual overlaps in the data. Therefore, we have chosen to retain the current design.
- To address the suggestion about focusing on the linear part of panel c, we have added a new panel (now Figure 4e) that highlights the linear relationship between neural and physical distances specifically for small physical distances.
- We have clarified the term “spatially shuffled” in the figure caption, explicitly defining it as “spatially shuffled ratemaps” in-text.

Reviewer #2, Comment 4:
The sentence “Notably, we find that for small physical distances, there is a…” could benefit from clarification. Does “small physical distances” refer to distances within the spacing of grid cells?
Response:
Thank you for pointing this out. With the addition of Figure 4e and the inclusion of physical units in Figure 4, we believe this should now be clearer. By “small physical distances,” we are referring to literal physical distances, not specifically distances within the spacing of grid cells.
Please let us know if further clarification or revisions are needed. Thank you for your time and consideration.
